# Liver Disease in Transaldolase Deficiency: Molecular Basis, Genotype–Phenotype Relationships, and Transplantation Outcomes—A Systematic Review

**DOI:** 10.3390/ijms27188100

**Published:** 2026-09-11

**Authors:** Patryk Lipiński, Piotr Socha, Irena Jankowska, Anna Tylki-Szymańska

**Affiliations:** 1 Third Department of Pediatrics, Center of Postgraduate Medical Education, 05-092 Dziekanów Leśny, Poland; 2 Department of Gastroenterology, Hepatology, Feeding Disorders and Pediatrics, The Children’s Memorial Health Institute, 04-730 Warsaw, Poland; p.socha@ipczd.pl (P.S.); i.m.jankowska@wp.pl (I.J.); 3 Department of Pediatrics, Nutrition and Metabolic Diseases, The Children’s Memorial Health Institute, 04-730 Warsaw, Poland; atylki@op.pl

**Keywords:** transaldolase deficiency, pentose phosphate pathway, genotype–phenotype relationship, polyols, seven-carbon sugars, hepatocellular carcinoma, liver transplantation

## Abstract

Transaldolase deficiency (TALDO-D) is an ultra-rare disorder of the non-oxidative pentose phosphate pathway with prominent liver disease and systemic accumulation of polyols and seven-carbon sugars. We systematically reviewed hepatic manifestations, molecular findings, genotype–phenotype relationships, and liver transplantation (LTx) experience. PubMed/MEDLINE and Europe PMC were searched from inception to 10 August 2026, with citation tracking; supplementary Scopus and Web of Science Core Collection searches were performed on 19 August and 1 September 2026, respectively. Twenty-nine primary clinical reports were included. Individual-patient reconciliation supported 51 genetically/biochemically confirmed patients as the primary analytic set; inclusion of one diagnostically uncertain patient with a homozygous in-frame *TALDO1* variant reported as a variant of uncertain significance produced an upper-bound sensitivity set of 52. Seven patients underwent LTx. *TALDO1* variants were heterogeneous, and intrafamilial and intragenotypic variability precluded reliable genotype-based prediction. LTx may provide organ-specific rescue for advanced liver disease, but persistent renal, endocrine, hematologic, and metabolic abnormalities require lifelong multisystem surveillance.

## 1. Introduction

Transaldolase deficiency (TALDO-D; OMIM 606003) is an autosomal recessive inborn error caused by biallelic pathogenic variants in *TALDO1*. Transaldolase is a key enzyme of the non-oxidative branch of the pentose phosphate pathway (PPP), catalyzing reversible transfer of three-carbon units between sugar phosphates. The PPP provides ribose-5-phosphate for nucleotide synthesis and generates reduced nicotinamide adenine dinucleotide phosphate (NADPH), which is required for reductive biosynthesis, maintenance of cellular redox homeostasis, and protection against oxidative stress.

These functions are especially relevant to the liver, which coordinates nutrient metabolism across fed and fasting states. Hepatic carbohydrate handling includes glycogen synthesis and breakdown, gluconeogenesis, and maintenance of systemic glucose availability; lipid metabolism includes fatty acid synthesis and oxidation, ketogenesis, cholesterol and lipoprotein metabolism, and bile acid production; amino acid catabolism is coupled to nitrogen disposal through the urea cycle. The liver also integrates micronutrient storage, xenobiotic biotransformation, plasma protein synthesis, and endocrine signaling. NADPH is required for reductive biosynthesis, cytochrome-associated detoxification, and regeneration of reduced glutathione. Consequently, disruption of pentose phosphate carbon recycling may affect both biosynthetic capacity and defense against oxidative or drug-induced stress. The first reported patient had liver cirrhosis and abnormal urinary polyol excretion, establishing TALDO-D as a pentose phosphate pathway disorder with a major hepatic phenotype [1].

The clinical spectrum has expanded substantially. The largest phenotype overview summarized clinical data from 34 patients, demonstrating frequent hepatomegaly, cytopenias, coagulopathy, renal disease, endocrine abnormalities, and high early mortality [2]. More recent long-term follow-up studies, including adult patients, have demonstrated that TALDO-D is associated with progressive liver fibrosis and cirrhosis, the need for liver transplantation (LTx), and survival into adulthood with persistent or progressive renal and endocrine complications [3,4].

Given the increasing number of reported liver transplantations in transaldolase deficiency, this systematic review aimed to characterize the hepatic phenotype of TALDO-D and to analyze published LTx cases, with particular attention to molecular findings and genotype–phenotype relationships.

## 2. Methods

This systematic review used data exclusively from previously published reports; therefore, ethics committee or institutional review board approval was not required.

This systematic review followed PRISMA 2020 principles [5]. The Joanna Briggs Institute (JBI) case-report and case-series checklists were used as structured assessments of reporting completeness, not as conventional risk-of-bias instruments [6]. They cannot resolve publication bias, selection bias, duplicate reporting, confounding, or the validity of pooled documentation frequencies. Item-level results are provided in Supplementary Table S5. No eligible report was excluded solely for incomplete reporting. We extracted diagnostic method, family structure, liver and extrahepatic manifestations, LTx indication and outcome, follow-up, immunosuppression, complications, and post-transplant biochemical data when reported.

PubMed/MEDLINE and Europe PMC were searched from inception through 10 August 2026. The PubMed/MEDLINE syntax was: (“transaldolase deficiency” OR “TALDO deficiency” OR *TALDO1* OR TALDO-D) AND (liver OR hepatic OR cholestasis OR cirrhosis OR hepatocellular OR transplant* OR renal OR kidney OR endocrine OR hypogonadism OR adulthood OR “long-term”). A supplementary Scopus search was performed on 19 August 2026. Web of Science Core Collection was searched on 1 September 2026 using the exact Smart Search query: “transaldolase deficiency” OR “TALDO deficiency” OR “TALDO-D” OR *TALDO1*. No date or language restrictions were applied. Reference lists and citing reports were checked when accessible. Conference abstracts were eligible only when they provided sufficient patient-level clinical data. Embase was not searched.

Eligible reports for the primary analysis described human TALDO-D supported by biallelic pathogenic/likely pathogenic *TALDO1* variants or a compatible pathway-specific biochemical profile and contained extractable clinical data. Reviews, non-human studies, unrelated *TALDO1* expression studies, and papers without clinical TALDO-D data were excluded. Variant nomenclature was harmonized to *TALDO1* transcript NM_006755.2 (protein NP_006746.1) when possible. One patient with homozygous c.871_873delGAG (p.Glu291del), reported as a variant of uncertain significance without published pathway-specific biochemical confirmation, was excluded from the primary set and retained only in an upper-bound sensitivity analysis (n = 52).

P.L. and P.S. independently screened records, assessed full texts, extracted data, and completed the reporting-completeness appraisal; disagreements were resolved by consensus. Bibliographic duplicates were removed before screening. Potentially repeated patients were reconciled using genotype, family structure, country/center, sex, age, phenotype, transplant details, and longitudinal course. The primary analytic set comprised 51 genetically or biochemically supported patients. Because limited public identifiers prevented definitive matching in every report, 51 remains an estimate rather than a census. A sensitivity analysis adding the diagnostically uncertain VUS case yielded an upper bound of 52 (Supplementary Table S4). Features were counted only when explicitly documented and are reported as minimum counts, not prevalence estimates.

## 3. Results

The original searches yielded 66 PubMed/MEDLINE records and 152 Europe PMC records; 11 additional records were found by citation tracking and full-text searching. After removal of 61 duplicate bibliographic records, 168 records were screened; 139 were excluded at title/abstract screening and 29 full-text primary clinical reports were included [1,2,3,4,7,8,9,10,11,12,13,14,15,16,17,18,19,20,21,22,23,24,25,26,27,28,29,30,31]. The supplementary Scopus update retrieved 94 records. The Web of Science Core Collection update retrieved 187 records (search date 1 September 2026); all exported titles and available abstracts were screened. Candidate clinical records represented reports already included, overlapping descriptions, conference abstracts without sufficient extractable patient-level information, or records outside the clinical scope, and no additional eligible primary report was added. Because both updates were performed after the original deduplicated workflow, they are displayed as separate update boxes in Supplementary Figure S1 rather than incorporated into an unverifiable revised cross-database duplicate count.

The structured JBI reporting-completeness assessment showed substantial variation among reports, particularly in longitudinal follow-up, intervention details, adverse-event reporting, consecutive or complete inclusion in series, and site descriptions (Supplementary Table S5). These findings describe transparency of reporting and should not be interpreted as proof of low risk of bias or validity of quantitative synthesis.

## 4. Hepatic Phenotype and Natural History

The 29 included reports support a primary analytic set of 51 genetically or biochemically confirmed patients. Supplementary Table S4 documents the patient-level linkage decisions and uncertainty. The Williams overview and Scaglione cohort substantially overlap with earlier reports and were not simply added together [2,3]. The diagnostically uncertain Asiri case with homozygous c.871_873delGAG (p.Glu291del) was excluded from primary analyses and retained only in an upper-bound sensitivity set of 52; its inclusion did not materially alter qualitative conclusions.

Clinical presentation was predominantly early, although the age at molecular or biochemical diagnosis varied widely. In the largest 34-patient overview, patients came to medical attention prenatally or between birth and 9 years of age (n = 33; one unknown; mean age 0.44 years). Of 34 patients, 22 (65%) presented prenatally or during the first month of life, whereas 12/34 presented after 3 months of age; one asymptomatic child was diagnosed at 9 years through family screening [2]. In the 16-patient long-term cohort, eight patients presented at birth, including six with neonatal liver failure, and the median age at diagnosis was 8 months (range 1 month–22 years) [3]. The adult natural history study further emphasized delayed recognition: three patients had neonatal hepatomegaly and cytopenias but were diagnosed only at 17, 26, and 32 years, respectively [4].

Consanguineous parentage was commonly reported, and several families had more than one affected child, as expected for an autosomal recessive disorder. In the Williams overview, 17 of 21 families (81%) were consanguineous and six families had more than one affected child [2]. In the Scaglione cohort, 12 of 16 patients came from consanguineous families, including five sibling pairs [3]. We did not calculate a single percentage for the pooled cohort because the largest overview reported consanguinity by family, while later cohorts included some patients described in earlier reports.

The phenotype was liver-predominant with multisystem involvement. In the primary confirmed set, a hepatic abnormality was documented in all 51 patients, but ascertainment and publication bias preclude interpretation as prevalence. Minimum documented counts were: hepatomegaly/hepatosplenomegaly, 42; cytopenia/thrombocytopenia, 37; anemia, 32; impaired coagulation, 14; portal hypertension, 15; fibrosis/cirrhosis, 16; renal involvement, 19; and endocrine/gonadal involvement, 11. Any cardiac abnormality was documented in at least 20 patients, including congenital heart defects or incidental PFO/PDA in at least 17. Impaired growth or growth failure was documented in at least eight patients. Three patients had confirmed HCC and seven putatively distinct patients underwent LTx. Adding the VUS case increased only selected descriptive counts by one (Supplementary Table S2).

Eight deaths were reported in the primary confirmed set. The cause of death was explicitly specified for part of this group: two infants in the long-term cohort died from severe liver failure before LTx was feasible because persistent weight below 4 kg precluded transplantation, one child died from post-transplant portal thrombosis, pulmonary infection and respiratory failure, and one transplanted child with decompensated cirrhosis, hepatopulmonary syndrome and HCC died from cardiac arrest on postoperative day 1 [2,3,22]. The remaining deaths were reported in earlier overlapping clinical summaries, most often in the context of severe early hepatic or multisystem disease, but individual terminal events were not consistently described in the included reports [2,3].

The long-term cohort reported by Scaglione et al. demonstrated both early severity and subsequent heterogeneity of hepatic disease. Hepatomegaly was universal and splenomegaly occurred in 15/16, while jaundice affected 9/16. With disease progression, 15/16 developed clinical portal hypertension; esophageal varices were identified in five and ascites in four. Progressive hepatic decompensation was reflected by prolonged coagulation parameters (prothrombin time < 70% in 12/16 patients) and micronodular cirrhosis in 10/16 [3].

Overall, the natural history ranged from fetal or neonatal lethal disease to slowly progressive chronic liver disease with survival into adulthood. In the 16-patient long-term cohort, 2/16 died before feasible LTx, 2/16 underwent early LTx, 2/16 underwent later LTx before age 20 years, and 10/16 followed a slower course without liver transplantation [3]. Adult cases showed that neonatal onset does not necessarily predict immediate severe outcome, but delayed diagnosis may allow renal, endocrine, bone and hepatic complications to accumulate over time [4].

Pediatric HCC and suspicious focal lesions have been reported [2,13,22]. Absolute risk cannot be estimated. In patients with advanced fibrosis, cirrhosis, portal hypertension, nodular disease, or changing alpha-fetoprotein, regular imaging and alpha-fetoprotein monitoring may be considered as an expert-practice suggestion. No validated TALDO-D-specific surveillance interval exists; timing should be individualized by a specialist hepatology team.

Biochemical diagnosis is based on accumulation of urinary and/or plasma polyols and seven-carbon sugars, including erythritol, D-arabitol, ribitol, sedoheptulose, sedoheptitol, perseitol, mannoheptulose, and phosphorylated intermediates when specialized methods are available [1,9,18,32]. Diagnostic pathways differed across the literature. Earlier patients were often recognized through abnormal urinary polyol profiles followed by *TALDO1* sequencing, whereas more recent patients are increasingly identified by exome, genome, or targeted liver disease gene panels and then supported by biochemical confirmation. In practice, suspected TALDO-D is now frequently established molecularly, but biochemical testing remains important for pathway confirmation, variant interpretation, family studies, and baseline monitoring. Conversely, mild or adult presentations may be missed if urinary polyols are not measured or if genomic testing is interpreted without awareness of TALDO-D.

## 5. Molecular Genetics and Genotype–Phenotype Relationships

TALDO-D is caused by biallelic pathogenic variants in *TALDO1*. The largest molecular overview available before the recent natural history cohorts identified 11 different *TALDO1* variants among 34 patients; 94% of patients were homozygous. The most frequent alleles were c.793delC (p.Gln265Argfs*56), c.512_514delCCT (p.Ser171del), and c.574C>T (p.Arg192Cys) [2]. The reported molecular spectrum includes missense, in-frame deletion, frameshift, truncating, splice site, and exon deletion variants, consistent with loss of transaldolase function.

Recent studies have expanded this allelic spectrum and, importantly, have provided longer longitudinal observation. The 2026 adult natural history study reported three genetically confirmed adults, including homozygous c.574C>T (p.Arg192Cys), compound heterozygosity for c.131_133del (p.Thr44del) and deletion of exon 3, and homozygous c.345dupA (p.Asp116Argfs*80) [4]. The 2026 multicenter long-term cohort included recurrent homozygous c.512_514delCCT and c.575G>A variants together with truncating, splice site, deletion, and other rare genotypes [3]. These data reinforce substantial allelic heterogeneity and show that molecular diagnosis may be delayed despite manifestations beginning in fetal life, the neonatal period, or infancy.

A simple genotype–phenotype relationship has not been established. Williams et al. observed that all five patients from two families homozygous for c.512_514delCCT (p.Ser171del) presented intrauterinely or neonatally, whereas three patients from one family homozygous for c.512C>T (p.Ser171Phe) had later-onset presentation [2]. However, marked intrafamilial and intragenotypic variability limits prognostic interpretation. In one family carrying p.Ser171Phe, siblings ranged from an apparently asymptomatic child to cirrhosis and HCC [2,13]. Likewise, patients sharing recurrent genotypes have shown different trajectories of hepatic and extrahepatic disease.

The 2026 long-term cohort provides additional evidence for both possible genotype-associated patterns and important exceptions [3]. Three patients sharing homozygous c.575G>A underwent LTx for different indications, arguing against a uniform hepatic trajectory. A sibling pair homozygous for c.926_927del had a very severe early course, whereas siblings with homozygous c.512_514delCCT shared a similar renal phenotype but differed in hepatic severity, with mild fibrosis in one and cirrhosis in the other. Conversely, a sibling pair with a homozygous exon 1 deletion showed a closely similar phenotype with early cirrhosis, severe hemorrhagic manifestations, adrenal insufficiency, and hypothyroidism. Thus, selected variants or family-specific genotypes may be associated with recognizable patterns, but the evidence remains insufficient for variant-based prediction of liver failure, HCC, or transplantation need.

**Figure 1:**
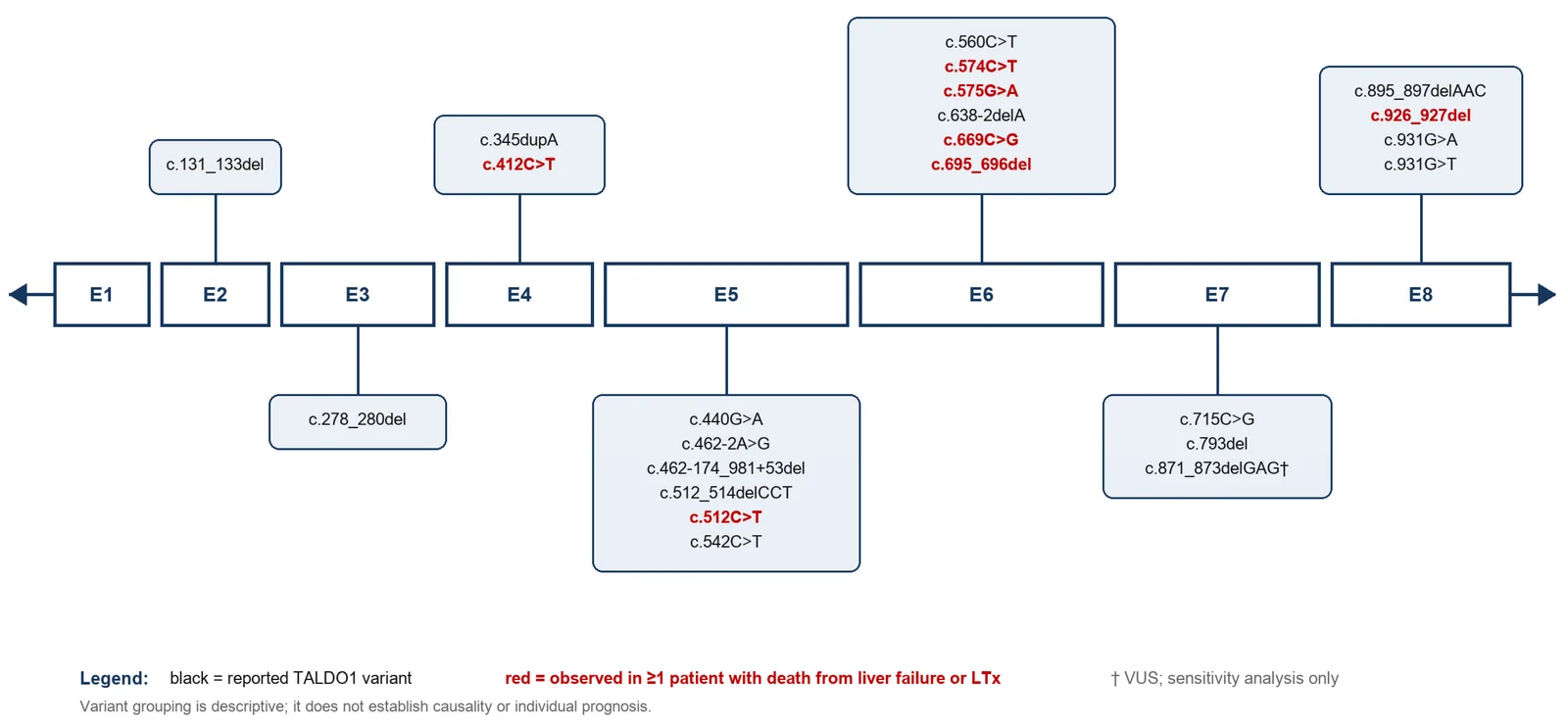
Reported *TALDO1* variants grouped by exon. Variants shown in red were observed in at least one patient with a severe hepatic outcome, defined descriptively as death from liver failure or liver transplantation. This marking is an observational visual aid only; it does not imply causality, establish a mutation-specific threshold, or predict the course of an individual patient. The c.871_873delGAG (p.Glu291del) variant was reported as a variant of uncertain significance and is retained only in the sensitivity presentation. The dagger symbol denotes a variant of uncertain significance included in the sensitivity analysis only.

Accordingly, genotype should currently be regarded primarily as an etiologic and family-diagnostic marker rather than an independent prognostic biomarker. Residual enzyme activity, modifier genes, tissue-specific metabolic effects, oxidative stress, environmental exposures, and other unrecognized factors may contribute to the broad clinical variability. The reported *TALDO1* genotypes and associated hepatic, extrahepatic, and transplantation phenotypes are summarized in Supplementary Table S1; because several publications describe overlapping patients or cohorts, genotype–phenotype interpretation requires careful comparison of overlapping case descriptions rather than simple summation of published cases. Two additional siblings reported in 2026 carried a novel homozygous *TALDO1* variant and both had chronic fibrotic liver disease with proteinuria, hypergonadotropic hypogonadism, dysmorphism, and skeletal abnormalities; the published abstract does not provide HGVS nomenclature, so the variant is not assigned a cDNA/protein position in Figure 1 [28]. The distribution of reported TALDO1 variants grouped by exon is shown in Figure 1.

## 6. Molecular and Mechanistic Basis of Multisystem Disease

Transaldolase catalyzes reversible transfer of a three-carbon unit between sedoheptulose-7-phosphate and glyceraldehyde-3-phosphate, producing erythrose-4-phosphate and fructose-6-phosphate in the non-oxidative pentose phosphate pathway. This reaction connects pentose phosphate intermediates with glycolysis and supports recycling of excess ribose-5-phosphate toward glucose-6-phosphate. Although transaldolase does not generate NADPH directly, impaired non-oxidative carbon recycling can restrict substrate return to the oxidative pentose phosphate pathway and thereby reduce effective NADPH availability. Carbon trapping also favors accumulation of sugar phosphates, polyols, and seven-carbon metabolites that constitute the characteristic biochemical signature of TALDO-D [1,9,18,32].

NADPH is required for regeneration of reduced glutathione, reductive biosynthesis, nitric oxide homeostasis, and antioxidant defense. Experimental *TALDO1* deficiency is associated with depletion of NADPH and glutathione, mitochondrial membrane potential abnormalities, altered mitochondrial mass and respiratory function, and increased susceptibility to oxidant injury. In Taldo1-deficient mice, these disturbances increase vulnerability to acetaminophen-induced liver failure and are associated with nodular liver disease and hepatocarcinogenesis; N-acetylcysteine reduced oxidative injury and prevented tumor development in this model [33]. Patient-derived cellular data and the persistent human polyol phenotype are directionally consistent with redox disequilibrium, but they do not establish a uniform quantitative relationship between a metabolite concentration and clinical severity.

Oaks et al. further defined the interaction between transaldolase deficiency and cytosolic aldose metabolism [34]. In experimental models, aldose reductase acted as a regulator of pentose phosphate carbon recycling: polyol production consumed NADPH and contributed to mitochondrial oxidative stress, acetaminophen susceptibility, and progression from cirrhosis to hepatocellular carcinoma. Aldose reductase inactivation reduced mitochondrial electron transport chain activity, reactive oxygen intermediate production, and hepatocarcinogenesis, but it intensified carbon trapping and growth restriction and did not restore all depleted pathway intermediates. Thus, aldose metabolism may have context-dependent effects—worsening NADPH depletion and oxidative injury while partly relieving accumulation of non-oxidative pentose phosphate intermediates. These results identify a plausible mechanism for hepatic progression but remain experimental and cannot yet be used to estimate HCC risk in an individual patient.

A complementary pathway was reported by Winans et al. [35]. In transaldolase-deficient experimental systems, mTOR activation was associated with reduced secretion of paraoxonase-1 (PON1), generation of antiphospholipid autoantibodies, and immune-mediated cirrhosis. PON1 has antioxidant and lipid-associated functions, and its loss may amplify oxidative and inflammatory injury. This mechanism offers a link between metabolic stress, altered hepatic secretory function, and autoimmunity that is not captured by a simple metabolite accumulation model. However, the contribution of this pathway to human TALDO-D, its relationship to genotype or age, and its potential value as a biomarker or therapeutic target require prospective validation.

**Figure 2:**
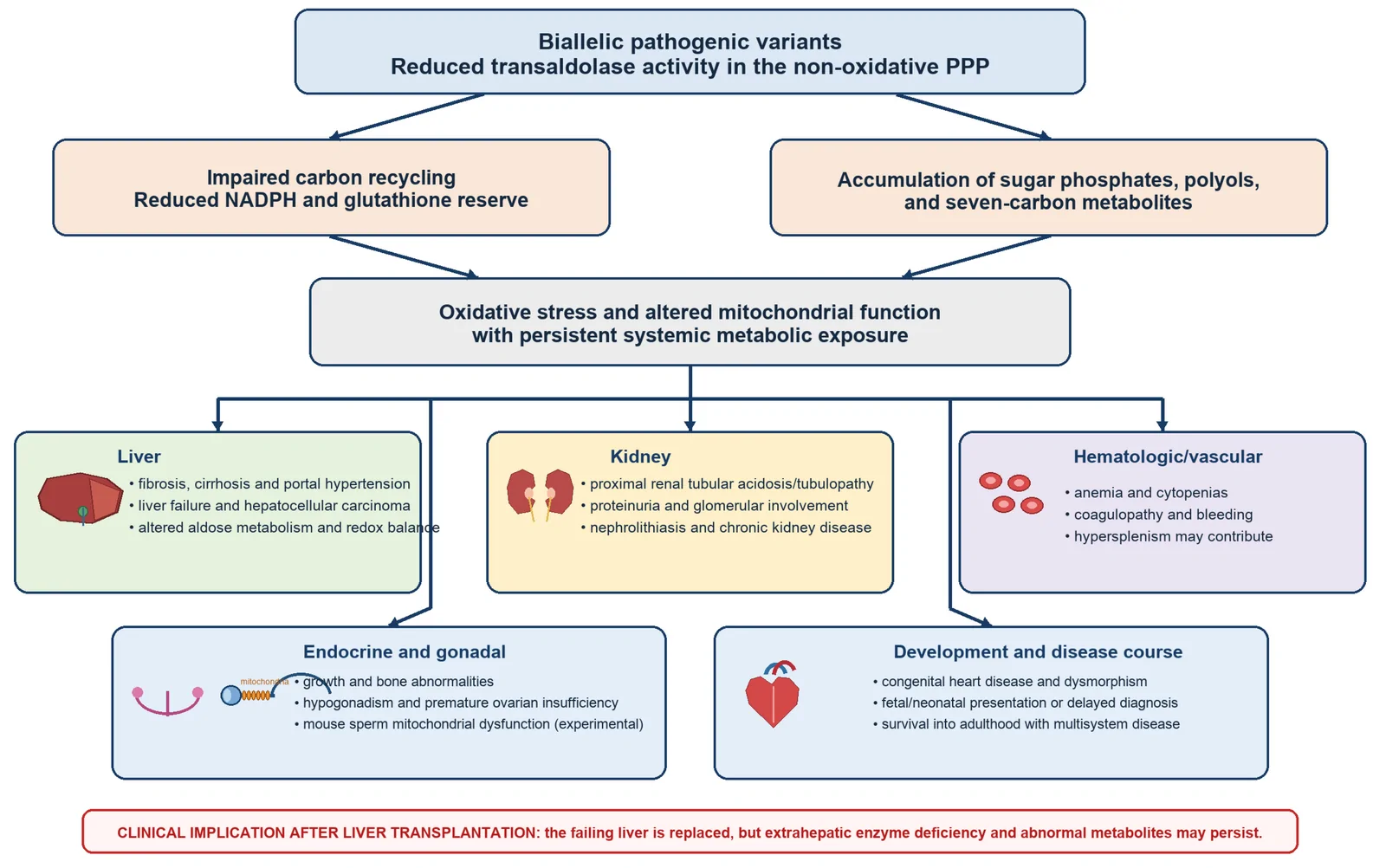
Proposed multisystem pathophysiology and clinical spectrum of transaldolase deficiency. Biallelic *TALDO1* deficiency disrupts carbon recycling in the non-oxidative pentose phosphate pathway, reduces nicotinamide adenine dinucleotide phosphate (NADPH) and glutathione reserve, and promotes the accumulation of polyols, seven-carbon sugars, and phosphorylated intermediates. The organ icons are schematic: hepatic, renal, hematologic/vascular, endocrine/gonadal, cardiac, developmental, and adult manifestations are summarized. The sperm inset denotes mitochondrial dysfunction demonstrated in *TALDO1*-deficient mice and should not be interpreted as an established mechanism of human gonadal disease [37]. The lower red-bordered box is a clinical post-transplantation qualification rather than a causal step: liver transplantation replaces the failing liver but does not correct *TALDO1* deficiency in extrahepatic tissues or necessarily normalize the systemic biochemical phenotype.

The same upstream redox and metabolite disturbances provide a conceptual framework for multisystem disease, but organ-specific causal chains remain incompletely resolved (Figure 2). Renal manifestations include proximal and distal tubular dysfunction, proteinuria, stones, glomerular involvement, and chronic kidney disease [3,4,12,30,36]. Endocrine and gonadal manifestations include growth and bone abnormalities, hypergonadotropic hypogonadism, and premature ovarian insufficiency [2,3,4,20,24,25,29]. *TALDO1*-deficient mice show reduced sperm mitochondrial membrane potential and motility with male infertility [37], but this experimental phenotype should not be assumed to explain human ovarian or testicular disease. Congenital heart disease, dysmorphism, cytopenias, and coagulopathy may reflect developmental effects, liver disease, portal hypertension, or tissue-specific metabolic vulnerability rather than a single shared downstream mechanism.

These mechanistic observations have therapeutic implications but do not establish an evidence-based disease-modifying treatment. N-acetylcysteine and other antioxidant strategies are biologically plausible and have limited case-based or experimental support [17,33], whereas pharmacological modulation of aldose reductase, mTOR, PON1-associated pathways, or immune mechanisms has not been evaluated clinically in TALDO-D. Liver transplantation restores transaldolase activity in donor hepatocytes and can correct hepatic failure, yet it does not correct *TALDO1* deficiency in the kidneys, gonads, hematopoietic cells, or other recipient tissues. Persistence of abnormal urinary metabolites after LTx therefore supports a multicompartment model of disease [13]. Future studies should combine serial metabolomics, redox biomarkers, renal and endocrine phenotyping, and graft outcomes to determine which pathways track clinical progression and which are merely associated experimental findings.

The proposed links between TALDO1 deficiency, disturbed non-oxidative pentose phosphate pathway flux, redox imbalance, metabolite accumulation, organ involvement, and the partial effect of LTx are summarized in Figure 2.

## 7. Liver Transplantation: Indications and Outcomes

Liver transplantation has been described in seven putatively distinct patients among the published upper-bound patient set [2,3,13,21,22,26]. Cross-linkage is summarized in Table 1 and Supplementary Table S4. The c.574C>T (p.Arg192Cys) Emirati child transplanted at 17 months corresponds to the Williams F17-P1 row; HCC was not attributed to this patient. The c.512C>T (p.Ser171Phe) Gambian child transplanted at 16 months had HCC. The three recipients in Grammatikopoulos et al. overlap with the Williams overview and/or the later Scaglione cohort and were not added again. The Williams personal communication case is treated as a probable match to the early post-transplant death later included by Scaglione, not as an additional recipient. The later Atasever case is retained separately. Residual uncertainty reflects incomplete public identifiers.

LTx replaces the major diseased organ and can restore hepatic synthetic function in survivors with functioning grafts, but it does not correct biallelic *TALDO1* deficiency in extrahepatic tissues. In the post-LTx urine sample reported by Leduc et al., ribitol was 54 [9,10,11,12,13,14,15,16,17,18,19,20,21,22,23,32], arabitol 152 (<89), sedoheptitol 2 (<1), sedoheptulose 600 (<9), sedoheptulose-7-phosphate 0.93 (<0.07), ribose-5-phosphate 0.76 (<0.13), and ribulose-5-phosphate plus xylulose-5-phosphate 0.84 (<0.44) mmol/mol creatinine [13]. These measurements directly demonstrate persistence of the metabolic signature after hepatic replacement.

Post-transplant outcome reporting was heterogeneous. Two of seven recipients died early after transplantation: one after portal thrombosis, pulmonary infection and respiratory failure, and one after postoperative cardiac arrest on day 1 [3,22]. Five of seven recipients were alive at the latest reported follow-up, with follow-up ranging from 1 year to adolescence or early adulthood when reported [3,13,21,26]. Functioning grafts were documented in the surviving recipients with available follow-up. Immunosuppression was described in three recipients: the two Polish brothers initially received tacrolimus, mycophenolate mofetil and transient steroids, with later conversion from tacrolimus to cyclosporine because of suspected nephrotoxicity [21], and Atasever et al. reported stable graft function at 1 year after LTx, with resolution of coagulopathy and recurrent bleeding and treatment of a biliary stricture during follow-up [26]. Post-LTx biochemical testing was reported in one recipient [13]. Renal and endocrine follow-up was sufficiently detailed only in selected surviving recipients; therefore, long-term extrahepatic outcomes after LTx remain incompletely characterized.

In summary, perioperative mortality was 2/7, survival at the latest reported follow-up was 5/7, and functioning graft status was documented in the surviving recipients with available follow-up. Follow-up ranged from 1 year to adolescence or early adulthood when reported. Immunosuppression was reported in 3/7 recipients, post-transplant biochemical testing in 1/7, and renal or endocrine follow-up in sufficient detail only in selected cases. These figures are crude descriptive proportions and not actuarial survival estimates.

**Table 1:** Liver transplantation in transaldolase deficiency: indications, biochemical phenotype, and reported follow-up.

Patient/Report	Genotype	Ltx Age/Indication	Outcome	Age/Duration at Last Follow-Up	Post-LTx Biochemistry	Immunosuppression/Complications	Ref.
Williams F13-P3	c.512C>T (p.Ser171Phe), hom.	~1 y; cirrhosis, HCC	Alive; functioning graft at ~7 y	~7 y of age	Abnormal urinary polyols/C7 sugars persisted	Not reported	[2,13]
Williams F17-P1/Emirati child	c.574C>T (p.Arg192Cys), hom.	17 mo; early-onset liver failure	Alive; normal graft function reported at 10 y; moderate failure to thrive and systemic hypertension	10 y of age	Not reported	Not reported	[2,15,23]
Williams personal communication/Scaglione early LTx death (probable match)	Not reported	5 mo; severe liver disease	Death 2 weeks after LTx	2 wk post-LTx (death)	Not reported	Portal thrombosis; pulmonary infection; respiratory failure	[2,3]
Grammatikopoulos P1	c.412C>T (p.Arg138*), hom.	11 mo; cirrhosis, HPS, nodules, HCC	Death on postoperative day 1	Postoperative day 1 (death)	Not reported	Cardiac arrest	[22]
Polish brother 1	c.575G>A (p.Arg192His), hom.	11 y; progressive cirrhosis/nodular liver	Alive; functioning graft	Not reported	Not reported	Tacrolimus + MMF + steroids; later cyclosporine	[3,21]
Polish brother 2	c.575G>A (p.Arg192His), hom.	14 y; cirrhosis; concern for malignancy	Alive; functioning graft	Not reported	Not reported	Tacrolimus + MMF + steroids; later cyclosporine	[3,21]
Atasever et al.	Not reported	3 y 4 mo; ESLD, bleeding, suspected HCC	Stable graft at 1 y; bleeding resolved	1 y post-LTx (~4 y 4 mo)	Not reported	Biliary stricture treated	[26]

Abbreviations: C7, seven-carbon; HCC, hepatocellular carcinoma; LTx, liver transplantation; MMF, mycophenolate mofetil. “Not reported” indicates that the cited publication did not provide the corresponding information. The post-LTx urinary metabolite measurements in Patient 1 provide direct evidence that the systemic biochemical phenotype can persist despite liver replacement [13].

## 8. Biochemical and Multisystem Outcomes After Liver Transplantation

The post-LTx biochemical question is central to interpreting transplantation in TALDO-D. The clearest published evidence shows persistence, rather than normalization, of the characteristic urinary metabolite profile after transplantation [13]. This finding separates correction of liver failure from correction of the systemic metabolic defect. Whether persistent exposure to polyols, seven-carbon sugars, phosphorylated intermediates, and an altered redox environment contributes to progressive injury in native extrahepatic organs or to secondary long-term injury of the genetically normal liver graft is unknown. No published report has demonstrated histological recurrence of TALDO-D in a liver allograft. Accordingly, recurrence should not be assumed; however, the absence of systematic long-term graft histology and serial metabolic profiling prevents this possibility from being fully assessed.

The timing of extrahepatic manifestations was variable (Supplementary Table S3). Cytopenias, congenital heart disease, dysmorphic or skin findings, growth restriction, and renal tubular abnormalities may coexist with hepatic disease from fetal life or infancy. Kidney involvement was present in 13/16 patients in Scaglione et al., tubulopathy/proteinuria in 11/16, and chronic kidney disease in 5/16 [3]. Earlier nephrology-focused data identified nephrological abnormalities in 7/9 patients [12]. Endocrine manifestations often became more apparent with longer survival: abnormal genitalia were reported in 11/34 and hypergonadotropic hypogonadism in 6/34 in Williams et al.; Scaglione et al. reported hypergonadotropic hypogonadism in 7/16 and adrenal/thyroid disease in 2/16 [2,3]. A 2025 case report described a 14-year-old girl with homozygous c.793del, type 1 diabetes, cirrhosis and portal hypertension, and severe proximal renal tubular acidosis documented by aminoaciduria, glucosuria, and phosphaturia; bicarbonate treatment improved the acidosis [36]. A separate 2025 report emphasized that hepatosplenomegaly and cytopenias may initially prompt evaluation for Gaucher disease, illustrating the importance of considering TALDO-D in the differential diagnosis [38].

## 9. Discussion

This review places the seven reported LTx recipients within the primary set of 51 confirmed patients and the upper-bound sensitivity set of 52. Four questions are most relevant for clinical interpretation: what drives hepatic progression, when LTx should be considered, what LTx corrects and fails to correct, and what surveillance is needed before and after transplantation.

What determines hepatic progression remains uncertain. The expanded mechanistic synthesis above identifies interacting redox, aldose metabolism, mitochondrial, mTOR/PON1, and immune pathways (Figure 2) [33,34,35,37]. These mechanisms are biologically coherent with the human hepatic phenotype, but most evidence is experimental, and none currently supports patient-level prediction from genotype, polyol burden, or a single biomarker.

The molecular data add an important qualification to this clinical synthesis. The reported *TALDO1* allelic spectrum is heterogeneous, while identical genotypes can be associated with markedly different hepatic and extrahepatic trajectories. This variability is consistent with TALDO-D being a systemic metabolic disorder in which the consequences of impaired non-oxidative pentose phosphate pathway flux, metabolite accumulation, and redox imbalance are likely modified by tissue-specific and environmental factors. At present, molecular diagnosis is therefore more robust for establishing etiology than for predicting the individual natural history.

A central finding is that TALDO-D does not follow a single natural history. The reported spectrum extends from fetal hydrops and fatal neonatal liver failure to compensated adult disease diagnosed decades after neonatal hepatomegaly and cytopenias [3,4,8]. Importantly, the adult natural history report emphasized that disease classification should be reconsidered because age at onset does not appear to determine severity [4]. This argues against a simple early- versus late-onset severity model and supports longitudinal risk assessment based on organ involvement, liver trajectory, portal hypertension, renal disease, endocrine complications, nutritional reserve, and family context. Consanguinity has been reported in some families, whereas other patients came from non-consanguineous families; the published literature is too ascertained to use consanguinity as a frequency estimate. However, recognition in consanguineous families and in siblings suggests that underdiagnosis is plausible, especially where urinary polyol testing and broad genomic diagnostics are not readily available. In neonates and infants, coagulopathy, cholestasis, hypoalbuminemia, failure to thrive, cytopenias, cardiomyopathy, and renal tubular dysfunction should prompt rapid metabolic and genomic evaluation. In older children and adolescents, TALDO-D may present with chronic cirrhosis, portal hypertension, splenomegaly, varices, growth failure, delayed puberty, renal disease, or focal liver lesions. Diagnosis in adulthood remains possible, especially when neonatal liver or hematologic abnormalities were incompletely explained.

When should LTx be considered? The reported indications were heterogeneous, including early liver failure, progressive cirrhosis and portal hypertension, hepatopulmonary syndrome or recurrent bleeding, and suspected or confirmed HCC [2,3,13,21,22,26]. This diversity argues against a single molecular signature for transplantation and supports individualized assessment based on the trajectory of liver disease rather than genotype alone. Early postoperative mortality in two recipients also emphasizes that LTx is a high-risk intervention in this fragile multisystem disease.

Accordingly, LTx should be considered an organ-specific rescue option for selected patients with life-threatening or end-stage hepatic disease after multidisciplinary evaluation in centers experienced in pediatric hepatology, metabolic disease, transplantation, and long-term multisystem follow-up.

What does LTx correct, and what does it not correct? LTx can correct liver failure and portal hypertension-related complications in survivors with functioning grafts, but it should not be framed as systemic metabolic cure. The strongest evidence is biochemical: a child with p.Ser171Phe TALDO-D had persistently abnormal urinary polyols, seven-carbon sugars, and phosphorylated sugars after LTx [13], and the larger biochemical overview concluded that urinary polyol excretion does not appear to normalize after transplantation [2]. A donor liver is expected to contain functional *TALDO1* alleles and transaldolase activity; donor genotyping was not reported, but the recipient’s kidneys and other tissues remain enzyme deficient. LTx therefore corrects the failing liver while leaving an extrahepatic source of abnormal metabolites and a persistent systemic biochemical phenotype.

What surveillance is required after transplantation? No recurrence of TALDO-D has been demonstrated histologically in a liver allograft, and true recurrence of the hepatocyte enzymatic defect would not be expected in genetically normal donor hepatocytes. Secondary graft injury related to a persistent systemic metabolic environment remains hypothetical and has not been proven. Comparisons with lysosomal acid lipase deficiency or methylmalonic acidemia should therefore be used only to illustrate the general distinction between organ replacement and systemic metabolic correction, not to imply a known risk of recurrent TALDO-D in the graft [39,40].

HCC risk remains difficult to quantify but clinically relevant [2,13,22]. The evidence is too sparse and ascertained to establish an evidence-based TALDO-D surveillance standard. Regular imaging and alpha-fetoprotein monitoring may be considered for advanced fibrosis, cirrhosis, or focal lesions, but no validated disease-specific interval exists and surveillance should be individualized.

For day-to-day pediatric hepatology practice, the most useful diagnostic clue is the coexistence of liver disease and extrahepatic manifestations. Cytopenias may reflect hypersplenism but can also precede overt portal hypertension. Renal tubular abnormalities, proteinuria or stones, abnormal genitalia, delayed puberty, osteopenia, and growth impairment should all increase suspicion. In this setting, biochemical testing for polyols/seven-carbon sugars and genomic testing including *TALDO1* are complementary rather than competing approaches.

This review has important limitations. Evidence consists of case reports, small series, overlapping cohorts, and retrospective descriptions. Patient-level reconciliation supports 51 confirmed patients, but incomplete identifiers prevent a definitive census; one VUS case is retained only in an upper-bound sensitivity set of 52. Organ systems were assessed inconsistently, so minimum counts cannot be interpreted as prevalence. Publication and survival bias are substantial. PubMed/MEDLINE, Europe PMC, Scopus, and Web of Science Core Collection were searched, but Embase was unavailable, so incomplete retrieval remains possible. The later Scopus and Web of Science updates could not be merged reproducibly into the original cross-database deduplication count and are shown separately. This review was not prospectively registered. JBI appraisal assessed reporting completeness rather than conventional risk of bias. These constraints limit genotype–phenotype inference and estimates of transplant outcomes.

Despite these limitations, the available data support several practical conclusions. First, TALDO-D should be considered in unexplained pediatric liver disease, particularly progressive fibrosis, cirrhosis, portal hypertension, or HCC, when hematologic, renal, or endocrine features coexist. Second, LTx evaluation is appropriate in selected patients with irreversible liver failure or advanced cirrhosis. Third, post-LTx care must remain multisystemic, because successful graft function does not remove the need for renal, endocrine, bone, growth, hematologic, cardiac, and biochemical surveillance. Serial urinary or plasma polyols and seven-carbon sugars may be considered for longitudinal characterization and research, but their prognostic value for graft outcome or extrahepatic progression remains undetermined.

## 10. Conclusions

TALDO-D is an ultra-rare multisystem disorder with a liver-predominant clinical phenotype. Patient-level reconciliation supports 51 confirmed patients; inclusion of one diagnostically uncertain VUS case gives an upper-bound sensitivity set of 52. Seven putatively distinct patients underwent LTx. No clinically reliable genotype–phenotype relationship predicts liver failure, HCC, or transplantation. The reported 5/7 survival is a crude proportion, not actuarial survival, and long-term renal and endocrine follow-up is incomplete. LTx should therefore be regarded as organ-specific rescue rather than systemic metabolic cure.

## Data Availability

All analyzed data were extracted from the cited publications. Supplementary Tables S1–S5 provide the report-level appraisal, variant summary, clinical documentation counts, and adjudicated patient-linkage decisions. The detailed extraction sheet and deduplication/linkage log are available from the corresponding author upon reasonable request.

## Supplementary Materials

The following supporting information can be downloaded at: https://www.mdpi.com/article/10.3390/ijms27188100/s1.

## References

[1] Verhoeven N.M., Huck J.H., Roos B., Struys E.A., Salomons G.S., Douwes A.C., van der Knaap M.S., Jakobs C. (2001). Transaldolase deficiency: Liver cirrhosis associated with a new inborn error in the pentose phosphate pathway. Am. J. Hum. Genet..

[2] Williams M., Valayannopoulos V., Altassan R., Chung W.K., Heijboer A.C., Keng W.T., Lapatto R., McClean P., Mulder M.F., Tylki-Szymańska A. (2019). Clinical, biochemical, and molecular overview of transaldolase deficiency and evaluation of the endocrine function: Update of 34 patients. J. Inherit. Metab. Dis..

[3] Scaglione M., Brassier A., Wiedemann A., Jankowska I., Pawłowska J., Lamireau T., Bridoux-Henno L., Douillard C., Casswall T., Cucinotta U. (2026). Long-term follow-up of patients with transaldolase deficiency. J. Inherit. Metab. Dis..

[4] Horvath V.B., Tsiakas K., Brennenstuhl H., Choukair D., Hammann N., Niesert M., Fichtner A., Prokisch H., Santer R., Wagner M. (2026). Transaldolase deficiency—Natural disease course towards adulthood. Mol. Genet. Metab..

[5] Page M.J., McKenzie J.E., Bossuyt P.M., Boutron I., Hoffmann T.C., Mulrow C.D., Shamseer L., Tetzlaff J.M., Akl E.A., Brennan S.E. (2021). The PRISMA 2020 statement: An updated guideline for reporting systematic reviews. BMJ.

[6] Joanna Briggs Institute (2020). Critical Appraisal Tools for Use in JBI Systematic Reviews: Checklist for Case Reports and Checklist for Case Series.

[7] Verhoeven N.M., Wallot M., Huck J.H.J., Dirsch O., Ballauf A., Neudorf U., Salomons G.S., van der Knaap M.S., Voit T., Jakobs C. (2005). A newborn with severe liver failure, cardiomyopathy and transaldolase deficiency. J. Inherit. Metab. Dis..

[8] Valayannopoulos V., Verhoeven N.M., Mention K., Salomons G.S., Sommelet D., Gonzales M., Touati G., de Lonlay P., Jakobs C., Saudubray J.-M. (2006). Transaldolase deficiency: A new cause of hydrops fetalis and neonatal multi-organ disease. J. Pediatr..

[9] Wamelink M.M., Struys E.A., Salomons G.S., Fowler D., Jakobs C., Clayton P.T. (2008). Transaldolase deficiency in a two-year-old boy with cirrhosis. Mol. Genet. Metab..

[10] Tylki-Szymańska A., Stradomska T.J., Wamelink M.M.C., Salomons G.S., Taybert J., Pawłowska J., Jakobs C. (2009). Transaldolase deficiency in two new patients with a relative mild phenotype. Mol. Genet. Metab..

[11] Balasubramaniam S., Wamelink M.M.C., Ngu L.H., Talib A., Salomons G.S., Jakobs C., Keng W. (2011). Novel heterozygous mutations in *TALDO1* gene causing transaldolase deficiency and early infantile liver failure. J. Pediatr. Gastroenterol. Nutr..

[12] Loeffen Y.G.T., Biebuyck N., Wamelink M.M.C., Jakobs C., Mulder M.F., Tylki-Szymańska A., Fung C.-W., Valayannopoulos V., Bökenkamp A. (2012). Nephrological abnormalities in patients with transaldolase deficiency. Nephrol. Dial. Transplant..

[13] Leduc C.A., Crouch E.E., Wilson A., Lefkowitch J., Wamelink M.M.C., Jakobs C., Salomons G.S., Sun X., Shen Y., Chung W.K. (2014). Novel association of early onset hepatocellular carcinoma with transaldolase deficiency. JIMD Report.

[14] Tylki-Szymańska A., Wamelink M.M.C., Stradomska T.J., Salomons G.S., Taybert J., Dąbrowska-Leonik N., Rurarz M. (2014). Clinical and molecular characteristics of two transaldolase-deficient patients. Eur. J. Pediatr..

[15] Al-Shamsi A.M., Ben-Salem S., Hertecant J., Al-Jasmi F. (2015). Transaldolase deficiency caused by the homozygous p.R192C mutation of the *TALDO1* gene in four Emirati patients with considerable phenotypic variability. Eur. J. Pediatr..

[16] Banne E., Meiner V., Shaag A., Katz-Brull R., Gamliel A., Korman S., Cederboim S.H., Duvdevani M.P., Frumkin A., Zilkha A. (2016). Transaldolase deficiency: A new case expands the phenotypic spectrum. JIMD Report.

[17] Rodan L.H., Berry G.T. (2017). N-acetylcysteine therapy in an infant with transaldolase deficiency is well tolerated and associated with normalization of alpha fetoprotein levels. JIMD Report.

[18] Lipiński P., Pawłowska J., Stradomska T., Ciara E., Jankowska I., Socha P., Tylki-Szymańska A. (2018). Long-term systematic monitoring of four Polish transaldolase deficient patients. JIMD Report.

[19] Lipiński P., Stradomska T., Tylki-Szymańska A. (2018). Transaldolase deficiency—Clinical outcome, pathogenesis, diagnostic process. Dev. Period Med..

[20] Lee-Barber J., English T.E., Britton J.F., Sobreira N., Goldstein J., Valle D., Bjornsson H.T. (2019). Apparent acetaminophen toxicity in a patient with transaldolase deficiency. JIMD Report.

[21] Lafcı N.G., Colak F.K., Sahin G., Sakar M., Çetinkaya S., Savas-Erdeve S. (2021). Hypergonadotrophic hypogonadism in a patient with transaldolase deficiency: Novel mutation in the pentose phosphate pathway. Hormones.

[22] Stefanowicz M., Janowska M., Pawłowska J., Tylki-Szymańska A., Kowalski A., Szymczak M., Kaliciński P., Jankowska I. (2021). Successful liver transplantation in two Polish brothers with transaldolase deficiency. Children.

[23] Grammatikopoulos T., Hadzic N., Foskett P., Strautnieks S., Samyn M., Vara R., Dhawan A., Hertecant J., Al Jasmi F., Rahman O. (2022). Liver disease and risk of hepatocellular carcinoma in children with mutations in *TALDO1*. Hepatol. Commun..

[24] Fallata E., Alamri A.M., Alrabee H.A., Alghamdi A.A., Alsaearei A. (2023). Chances of liver transplantation in a patient with transaldolase deficiency complicated by hepatopulmonary syndrome. Cureus.

[25] Takaleh A., Abunamous N., AlShamsi A., Alhassani N., Almazrouei R. (2024). Hypergonadotropic hypogonadism due to transaldolase deficiency: Two cases and literature review. JCEM Case Rep..

[26] Yıldız M., Önal Z., Yeşil G., Kabil T.G., Toksoy G., Poyrazoğlu Ş., Baş F., Durmaz Ö., Darendeliler F. (2025). A rare cause of hypergonadotropic hypogonadism: Transaldolase deficiency in two siblings. J. Clin. Res. Pediatr. Endocrinol..

[27] Atasever A., Yazici S.E., Turan E., Guller D., Yuzer Y. (2025). Successful liver transplantation in a pediatric patient with transaldolase deficiency. Pediatr. Transplant..

[28] Asiri K., Rayees S., Alasmari B.G. (2025). Transaldolase deficiency in a Saudi girl: Identification of a novel homozygous *TALDO1* variant. Cureus.

[29] Sumer Cosar O., Kayhan G., Akyol G., Dalgıç B. (2026). A rare cause of genetic liver disease in children: Transaldolase deficiency with a novel pathogenic variant in two siblings. Pediatr. Dev. Pathol..

[30] Köprülü Ö., Er E. (2026). A rare metabolic cause of premature ovarian insufficiency: Case report of transaldolase deficiency. J. Pediatr. Endocrinol. Metab..

[31] Gurbanova L., Büyükkaragöz B., Dalgic B. (2026). A rare cause of kidney disease in two siblings: Transaldolase deficiency. J. Nephrol..

[32] Xue J., Han J., Zhao X., Zhen L., Mei S., Hu Z., Li X. (2022). Prenatal diagnosis of fetus with transaldolase deficiency identifies compound heterozygous variants: A case report. Front. Genet..

[33] Hanczko R., Fernandez D.R., Doherty E., Qian Y., Vas G., Niland B., Telarico T., Garba A., Banerjee S., Middleton F.A. (2009). Prevention of hepatocarcinogenesis and increased susceptibility to acetaminophen-induced liver failure in transaldolase-deficient mice by N-acetylcysteine. J. Clin. Investig..

[34] Bernstein D.L., Lobritto S., Iuga A., Remotti H., Schiano T., Fiel M.I., Balwani M. (2018). Lysosomal acid lipase deficiency allograft recurrence and liver failure-clinical outcomes of 18 liver transplantation patients. Mol. Genet. Metab..

[35] Minnee R.C., Sakamoto S., Fukuda A., Uchida H., Hirukawa K., Honda M., Okumura S., Ito T., Yilmaz T.U., Fang Y. (2024). Long-term outcomes of living donor liver transplantation for methylmalonic acidemia. Pediatr. Transplant..

[36] Oaks Z., Patel A., Huang N., Choudhary G., Winans T., Faludi T., Krakko D., Duarte M., Lewis J., Beckford M. (2023). Cytosolic aldose metabolism contributes to progression from cirrhosis to hepatocarcinogenesis. Nat. Metab..

[37] Winans T., Oaks Z., Choudhary G., Patel A., Huang N., Faludi T., Krakko D., Nolan J., Lewis J., Blair S. (2023). mTOR-dependent loss of PON1 secretion and antiphospholipid autoantibody production underlie autoimmunity-mediated cirrhosis in transaldolase deficiency. J. Autoimmun..

[38] Perl A., Qian Y., Chohan K.R., Shirley C.R., Amidon W., Banerjee S., Middleton F.A., Conkrite K.L., Barcza M., Gonchoroff N. (2006). Transaldolase is essential for maintenance of the mitochondrial transmembrane potential and fertility of spermatozoa. Proc. Natl. Acad. Sci. USA.

[39] AlAkeel H., AlZaben A., AlAtawi M., Aldubayee M., AlEyaid W. (2025). A challenging metabolic acidosis management case in a young patient with transalodase defeciency, T1DM, and pRTA. J. Biochem. Clin. Genet..

[40] Panchal K., Antony S., Gawde N., Gangopadhyay S., Koyande S., David J.J.E., Vaswani R.K., Mundle D. (2025). Transaldolase (TALDO) deficiency—An under recognized Gaucher mimic. Indian J. Pediatr..

