# Supplementary material for: Liver Disease in Transaldolase Deficiency: Molecular Basis, Genotype–Phenotype Relationships, and Transplantation Outcomes—A Systematic Review
Source: Int J Mol Sci. 2026 Sep 11;27(18):8100. doi: 10.3390/ijms27188100 (PMC13606807; doi:10.3390/ijms27188100)
Supplement: Supplementary file 1 [file ijms-27-08100-s001.zip › TALDO_IJMS_Supplementary_Figure_S1.pdf]

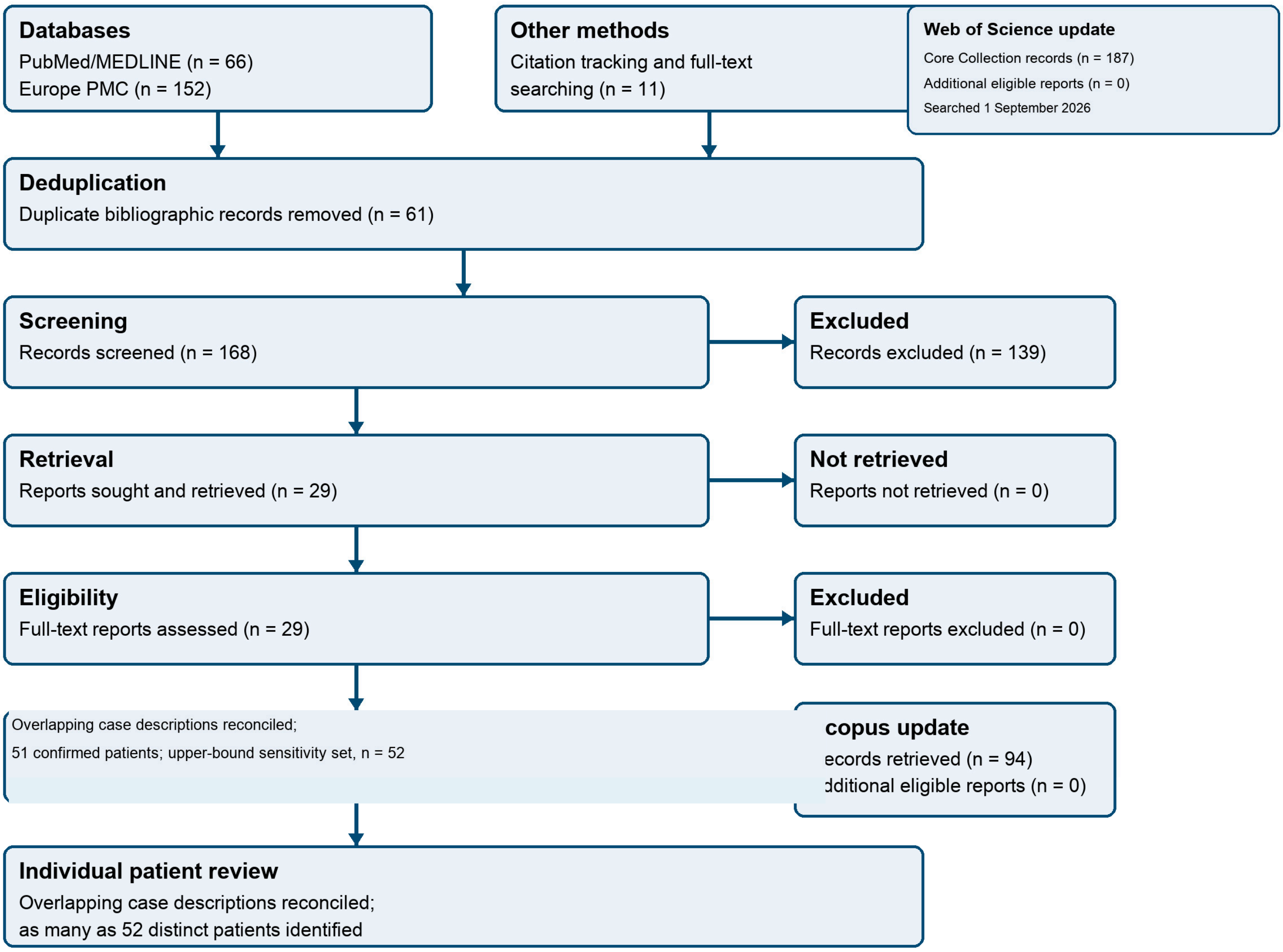

Supplementary Figure S1. PRISMA 2020 flowchart of study selection. The Scopus and Web of Science Core Collection searches were performed as later updates and are displayed separately because they were not merged into the original cross-database deduplication workflow.
