# Supplementary material for: Liver Disease in Transaldolase Deficiency: Molecular Basis, Genotype–Phenotype Relationships, and Transplantation Outcomes—A Systematic Review
Source: Int J Mol Sci. 2026 Sep 11;27(18):8100. doi: 10.3390/ijms27188100 (PMC13606807; doi:10.3390/ijms27188100)
Supplement: Supplementary file 1 [file ijms-27-08100-s001.zip › TALDO_IJMS_Supplementary_Table_S1.pdf]

Supplementary Table S1. Reported TALDO1 genotypes and associated hepatic, extrahepatic, and liver transplantation phenotypes.

TALDO1 variant (HGVS c.)	Protein change	Genotype / zygosity	Patient(s) / family	Hepatic phenotype (including HCC)	Extrahepatic phenotype	LTx	Reference(s)
c.512_514delCCT	p.Ser171del	Homozygous; recurrent	Five patients in the Williams overview; repeated descriptions were recognized when sufficient clinical and molecular details were available	Predominantly intrauterine/neonatal presentation; hepatomegaly/liver dysfunction; fibrosis/cirrhosis reported; variable long-term hepatic severity; no confirmed HCC assigned to this genotype in reviewed reports	Cytopenias; cardiac defects; renal disease including nephrocalcinosis/renal failure; genital abnormalities in some	No LTx directly assigned in source set used for this row	[1–3]
c.512C>T	p.Ser171Phe	Homozygous	3 siblings (Gambian family)	Late-onset/variable hepatic phenotype; hepatosplenomegaly; cirrhosis in affected siblings; early HCC in one; marked intrafamilial variability	Renal involvement reported in one sibling; otherwise comparatively mild extrahepatic phenotype	Yes — HCC-affected sibling underwent LTx in linked literature	[2,13]
c.574C>T	p.Arg192Cys	Homozygous; recurrent	Arab/UAE cases; adult case	Highly variable: hepatosplenomegaly/liver dysfunction to acute-on-chronic failure and cirrhosis; slow adult course also reported	Cytopenias, proteinuria/renal disease, cardiac and skin findings, growth impairment	Yes — Emirati child underwent LTx at 17 months for early liver failure; no HCC attributed to this patient	[2,4,15,22]
c.575G>A	p.Arg192His	Homozygous; recurrent	Polish brothers; 2026 cohort	Early liver injury; progressive nodular fibrosis/cirrhosis and portal hypertension; suspected HCC in one but not confirmed	Cytopenias, renal tubulopathy, growth impairment, skin changes; cryptorchidism	Yes — multiple patients; Polish brothers at 11 and 14 y; another early LTx	[3,18,21]
c.793del	p.Gln265Argfs*56	Homozygous; founder	12 Saudi patients/6 families; later HPS case	Hepatosplenomegaly/liver dysfunction common; advanced liver disease/portal hypertension reported; no confirmed HCC in linked reports	Growth retardation, dysmorphism/cutis laxa, congenital heart disease, pancytopenia/bleeding; HPS	No completed LTx confirmed; transplant evaluation reported	[2,23]
c.669C>G	p.Tyr223*	Homozygous	Single infant	Liver dysfunction with hepatosplenomegaly; severe early multisystem disease; no HCC reported	IUGR, dysmorphism, cytopenia, cardiac and renal involvement	No reported LTx	[16]
c.895_897delAAC + c.931G>A	p.Asn299del + p.Gly311Arg	Compound heterozygous	Single child	Hepatosplenomegaly with coagulopathy/cytopenias; no HCC reported	Dysmorphism, cardiac/respiratory findings, neonatal edema	No reported LTx	[11]
c.512_514delCCT + c.931G>T	p.Ser171del + p.Gly311Trp	Compound heterozygous	Single US child	Early hepatic disease; apparent acetaminophen-associated acute worsening; no HCC reported	IUGR/dysmorphism; thrombocytopenia; respiratory involvement	No reported LTx	[19]
c.462-174_981+53del	p.?	Homozygous	Polish patient	Early hepatosplenomegaly/liver dysfunction; progressive nodular fibrosis; no HCC	Renal involvement, osteopenia, poor growth	No reported LTx	[14,18]
c.575G>A + c.462-174_981+53del	p.Arg192His + p.?	Compound heterozygous	Polish patient	Early hepatosplenomegaly/liver dysfunction; nodular fibrosis; no HCC	Nephrolithiasis/tubular abnormalities, osteopenia, poor growth, genital abnormality	No reported LTx	[14,18]
c.715C>G	p.Arg239Gly	Homozygous	Single Turkish patient	Hepatosplenomegaly; no HCC described	Renal involvement; abnormal genitalia/hypergonadotropic hypogonadism	No reported LTx	[20]
c.412C>T	p.Arg138*	Homozygous	Grammatikopoulos Patient 1	Severe early liver disease; HCC reported in 2022 liver-focused series	Multisystem involvement	Yes — transplanted; HCC confirmed	[22]
c.542C>T + c.560C>T	p.Ala181Val + p.Ser187Phe	Compound heterozygous	Grammatikopoulos Patient 2	Chronic liver disease with multiple dysplastic nodules; no confirmed HCC at follow-up	Extrahepatic manifestations reported in source	No LTx at reported follow-up	[22]
c.695_696del	p.Ile232Serfs*19	Homozygous	Grammatikopoulos Patient 3	Subacute liver failure; cirrhosis, steatosis and cholestasis; no HCC in explant	Growth/neurodevelopmental issues; later renal dysfunction and premature ovarian insufficiency	Yes — LTx at 5 months	[22]
c.462-2A>G + c.574C>T	splice + p.Arg192Cys	Compound heterozygous	Chinese prenatal/neonatal case	Prenatal/neonatal hepatosplenomegaly, liver dysfunction and coagulopathy; no HCC	Fetal/neonatal anemia, dysmorphism, renal involvement	No reported LTx	[31]
c.638-2delA	splice-site	Homozygous	Scaglione Patient 7	Cirrhosis and portal hypertension with slow course; no HCC	Kidney involvement/CKD	No LTx	[3]
c.926_927del	frameshift	Homozygous; sibling pair	Scaglione Patients 8–9	Very severe early liver disease; one early LTx, one death; no HCC	Severe multisystem disease; early kidney disease in one	Yes — one sibling; post-LTx death	[3]
Exon 1 deletion (breakpoints/HGVS	p.?	Homozygous; sibling pair	Scaglione Patients 10–11	Early-onset cirrhosis; no HCC	Hemorrhagic manifestations; adrenal insufficiency, hypothyroidism; CKD	No LTx	[3]

TALDO1 variant (HGVS c.)	Protein change	Genotype / zygosity	Patient(s) / family	Hepatic phenotype (including HCC)	Extrahepatic phenotype	LTx	Referenc e(s)
coding description not reported in reviewed source)							
c.440G>A + c.278_280del	protein change not reported	Compound heterozygous	Scaglione Patient 12	Mild hepatic phenotype (F0); portal-hypertension signs; no HCC	Early renal tubulopathy/proteinuria	No LTx	[3]
c.131_133del + exon 3 deletion	p.Thr44del + p.?	Compound heterozygous	Adult natural-history Patient 2	Neonatal hepatic manifestations with survival to adulthood; slow progression; no HCC	Adult renal and endocrine involvement	No reported LTx	[4]
c.345dupA	p.Asp116Argfs*80	Homozygous	Adult natural-history Patient 3	Neonatal hepatic manifestations with survival to adulthood; no HCC	Adult renal/endocrine involvement and cytopenias	No reported LTx	[4]
c.871_873delGAG †	p.Glu291del	Homozygous; reported as a variant of uncertain significance in the source report	Saudi girl (included on clinical grounds; VUS status retained) (included on clinical grounds; VUS status retained)	Hepatomegaly, fibrotic changes and persistent focal lesion; no HCC confirmed	Failure to thrive, dysmorphism, congenital heart disease; mild renal finding	No reported LTx; excluded in the additional analysis using 51 patients	[27]
Novel homozygous *TALDO1* variant; exact HGVS coding designation not available in the publicly accessible abstract	Not reported in indexed abstract	Homozygous; sibling pair	Two Turkish siblings reported in 2026	Chronic liver disease with periportal/bridging fibrosis; hepatosplenomegaly in index case; 6-mm focal lesion in sibling under HCC surveillance; no malignant transformation on biopsy	Dysmorphic facial features, hypergonadotropic hypogonadism, proteinuria, scoliosis/skeletal abnormalities	No LTx reported	[28]

Note. Variant nomenclature is harmonized to TALDO1 transcript NM_006755.2 (NP_006746.1) where possible. Exon-level deletions without breakpoint data are reproduced as author-reported descriptions and are not fully HGVS validated. c.871_873delGAG (p.Glu291del) was reported using NM_006755.1 as a VUS without published pathway-specific biochemical confirmation. The patient was included after clinical adjudication because the phenotype was highly characteristic of TALDO-D; molecular uncertainty is retained and sensitivity analysis excludes this case (n = 51).
