# Supplementary material for: Liver Disease in Transaldolase Deficiency: Molecular Basis, Genotype–Phenotype Relationships, and Transplantation Outcomes—A Systematic Review
Source: Int J Mol Sci. 2026 Sep 11;27(18):8100. doi: 10.3390/ijms27188100 (PMC13606807; doi:10.3390/ijms27188100)
Supplement: Supplementary file 1 [file ijms-27-08100-s001.zip › TALDO_IJMS_Supplementary_Table_S2.pdf]

Supplementary Table S2. Documented clinical characteristics, family-history data, and outcomes in reported TALDO-D patients.

| Domain | Manifestation/outcome | Count | Assessed denominator | Comment |
|---|---|---|---|---|
| Overall | Unique reported patients represented in the reviewed literature | 52 | All 52 reported patients | The 52 patients include the VUS case and two subsequently reported siblings. Excluding the VUS case reduces the number to 51. |
| Hepatic | Liver involvement | 52 | Not recoverable | Includes neonatal liver failure, cholestasis, hepatomegaly/hepatosplenomegaly, chronic liver disease, fibrosis/cirrhosis, portal hypertension, focal lesions, HCC, or LTx. |
| Hepatic | Hepatomegaly or hepatosplenomegaly | 43 | Not recoverable | Minimum documented count; source reports did not uniformly state whether all patients were assessed. |
| Hepatic | Impaired coagulation/coagulopathy | 14 | Not recoverable | Documented as impaired coagulation, bleeding tendency related to liver disease, or liver synthetic dysfunction. |
| Hepatic | Portal hypertension | 15 | Not recoverable | Includes clinical portal hypertension and its complications. |
| Hepatic | Fibrosis or cirrhosis | 16 | Not recoverable | Includes two subsequently reported siblings with chronic fibrotic liver disease. |
| Hepatic/oncologic | Confirmed hepatocellular carcinoma | 3 | Not recoverable | Only confirmed HCC was counted. |
| Hepatic/oncologic | Focal liver lesion requiring HCC surveillance without malignant transformation | 1 | Not recoverable | Recently reported child with a small focal liver lesion and no malignant transformation on biopsy. |
| Hematologic | Thrombocytopenia or another cytopenia | 37 | Not recoverable | Includes thrombocytopenia and broader cytopenic phenotypes. |
| Hematologic | Anemia | 32 | Not recoverable | Anemia documented in individual reports or cohort-level summaries. |
| Hematologic | Pancytopenia | 5 | Not recoverable | Counted when specifically reported. |
| Renal | Any renal involvement | 19 | Not recoverable | Includes tubulopathy, proteinuria, hypercalciuria, nephrolithiasis, reduced renal reserve, or CKD. |
| Renal | Tubulopathy and/or proteinuria | 13 | Not recoverable | Includes two subsequently reported siblings with proteinuria. |
| Renal | Renal stones/nephrolithiasis | 4 | Not recoverable | Counted when explicitly reported. |
| Renal | Reduced renal reserve or chronic kidney disease | 5 | Not recoverable | Counted when explicitly reported. |
| Endocrine/gonadal | Any endocrine or gonadal involvement | 11 | Not recoverable | Includes hypergonadotropic hypogonadism and adrenal/thyroid disease. |
| Endocrine/gonadal | Hypergonadotropic hypogonadism | 8 | Not recoverable | Includes two subsequently reported siblings with hypergonadotropic hypogonadism. |
| Endocrine/gonadal | Adrenal or thyroid disease | 2 | Not recoverable | Counted when explicitly reported. |
| Growth/skeletal | Impaired growth or growth failure | 9 | Not recoverable | Counted when explicitly reported. |
| Growth/skeletal | Skeletal abnormalities | 2 | Not recoverable | Includes two subsequently reported siblings with skeletal abnormalities. |
| Cardiac | Any cardiac abnormality | 21 | Not recoverable | Includes congenital heart defects, PFO/PDA, ventricular hypertrophy/dysfunction, or cardiomyopathy reported in the linked literature. |
| Cardiac | Congenital heart defects/PFO/PDA | 18 | Not recoverable | Grouped because reports used variable cardiac terminology. |
| Cardiac | Cardiomyopathy or ventricular dysfunction/hypertrophy | 5 | Not recoverable | Includes cardiomyopathy, ventricular dysfunction, or ventricular hypertrophy when explicitly reported. |
| Outcome | Death during reported follow-up | 8 | Not recoverable | Deaths reported in the included published reports. |
| Treatment/outcome | Liver transplantation | 7 | Not recoverable | Patients reported to undergo LTx after review of overlapping reports. |

| Domain | Manifestation/outcome | Count | Assessed denominator | Comment |
|---|---|---|---|---|
| Clinical context | Median age at diagnosis in long-term cohort | 16 patients | Not recoverable | Source-specific denominator; cohort overlaps with earlier reports and was not added as independent patients. |
| Clinical context | Presentation at birth in long-term cohort | 8 | Not recoverable | Includes 6/16 with neonatal liver failure. |
| Clinical context | Adult diagnosis after neonatal manifestations | 3 | Not recoverable | Diagnoses at 17, 26 and 32 years despite neonatal hepatomegaly and cytopenias. |
| Family history | Consanguineous families in largest overview | 17 | Not recoverable | Family-level statistic from Williams et al.; not additive with overlapping cohorts. |
| Family history | Consanguineous families in long-term cohort | 12 | Not recoverable | Includes five sibling pairs. |
| Outcome | Death with severe liver failure before feasible LTx | 2 | Not recoverable | Both infants had persistent weight below 4 kg, precluding LTx. |
| Outcome | Death from early post-LTx complications | 2 | Not recoverable | One death followed portal thrombosis, pulmonary infection and respiratory failure; one occurred after postoperative cardiac arrest. |
| Outcome | Death with incompletely specified terminal event in included reports | 4 | Not recoverable | Reported in earlier overlapping summaries, usually in severe early hepatic or multisystem disease. |

Note. The available reports appear to describe 51 confirmed patients (52 in the upper-bound sensitivity analysis). Values are the minimum numbers in whom each feature was documented. The number actually assessed was usually unavailable; therefore, non-reporting is unknown rather than absence, and percentages are not presented as prevalence. The c.871_873delGAG VUS case was included because the reported phenotype was highly characteristic of TALDO-D; molecular uncertainty is retained. Excluding that patient reduced the number to 51 and did not materially change the qualitative conclusions. Counts were derived after repeated descriptions of the same patient had been identified wherever the published information allowed.
