# Supplementary material for: Liver Disease in Transaldolase Deficiency: Molecular Basis, Genotype–Phenotype Relationships, and Transplantation Outcomes—A Systematic Review
Source: Int J Mol Sci. 2026 Sep 11;27(18):8100. doi: 10.3390/ijms27188100 (PMC13606807; doi:10.3390/ijms27188100)
Supplement: Supplementary file 1 [file ijms-27-08100-s001.zip › TALDO_IJMS_Supplementary_Table_S3.pdf]

Supplementary Table S3. Extrahepatic manifestations relevant to transplant evaluation and long-term follow-up.

System	Reported manifestations	Relevance to LTx	Suggested monitoring
Hematologic	Anemia, thrombocytopenia, pancytopenia, and bleeding tendency.	May improve when driven by portal hypertension but may persist when related to systemic disease.	Complete blood count, coagulation profile, spleen size, and bleeding history.
Renal	Tubulopathy, hypercalciuria, nephrolithiasis, proteinuria, and chronic kidney disease.	A major determinant of perioperative risk and long-term outcome.	Creatinine/cystatin C, electrolytes, bicarbonate, urinalysis, urine calcium/creatinine ratio, and renal ultrasonography.
Endocrine	Hypergonadotropic hypogonadism, delayed puberty, premature ovarian insufficiency, hypothyroidism, and adrenal insufficiency.	Not expected to be corrected by LTx; affects growth, fertility, and bone health.	LH, FSH, sex steroids, thyroid function, cortisol when indicated, and specialist follow-up.
Bone and growth	Intrauterine growth restriction, postnatal growth failure, osteopenia, and low bone mineral density.	Nutritional reserve and frailty may affect transplant candidacy and recovery.	Anthropometry, vitamin D and mineral status, DXA when appropriate, and dietetic assessment.
Cardiac/pulmonary	Incidental PFO/PDA reported separately from clinically significant structural heart disease, ventricular dysfunction/hypertrophy, cardiomyopathy, and hepatopulmonary syndrome.	Relevant to anesthesia, perioperative management, and transplant risk.	ECG, echocardiography, oxygen saturation, and contrast echocardiography when hepatopulmonary syndrome is suspected.
Neurologic/developmental	Hypotonia, motor delay, or mild intellectual disability in some patients.	Usually not the dominant contraindication to LTx but may influence rehabilitation needs.	Developmental and neurologic assessment with rehabilitation support.

Abbreviations: DXA, dual-energy X-ray absorptiometry; ECG, electrocardiography; FSH, follicle-stimulating hormone; LH, luteinizing hormone; LTx, liver transplantation.
