# Supplementary material for: Liver Disease in Transaldolase Deficiency: Molecular Basis, Genotype–Phenotype Relationships, and Transplantation Outcomes—A Systematic Review
Source: Int J Mol Sci. 2026 Sep 11;27(18):8100. doi: 10.3390/ijms27188100 (PMC13606807; doi:10.3390/ijms27188100)
Supplement: Supplementary file 1 [file ijms-27-08100-s001.zip › TALDO_IJMS_Supplementary_Table_S4_patient_cross_reference.pdf]

Supplementary Table S4. Comparison of reports describing the 51 confirmed patients (52 in the upper-bound sensitivity analysis) identified in the literature.

Note. Reports were compared using the available information on family, country or center, genotype, sex, age, clinical features, transplantation, and follow-up. Some descriptions could not be matched with certainty because individual case details were limited. The patient with c.871_873delGAG (p.Glu291del) was included as patient 52 because the clinical picture was highly characteristic of TALDO-D; the variant nevertheless remains a VUS. Excluding this case reduces the number to 51.

Review ID	Primary publication	Overlapping/follow-up publications	Family/pedigree identifier	Genotype	Sex / age information	Information suggesting the same patient	Interpretation
P01	Williams et al., 2019	Earlier reports and later Scaglione/Horvath cohorts when matching details were available	Williams overview patient; source family/pedigree label when reported	As reported in source overview; genotype group summarized in Supplementary Table 1	Age/sex not uniformly extractable from overlapping summaries	The Williams overview could not be matched patient by patient with all earlier or later reports because individual case details were limited.	Included among the 34 patients summarized by Williams et al.; possible overlap with other reports remains uncertain.
P02	Williams et al., 2019	Earlier reports and later Scaglione/Horvath cohorts when matching details were available	Williams overview patient; source family/pedigree label when reported	As reported in source overview; genotype group summarized in Supplementary Table 1	Age/sex not uniformly extractable from overlapping summaries	The Williams overview could not be matched patient by patient with all earlier or later reports because individual case details were limited.	Included among the 34 patients summarized by Williams et al.; possible overlap with other reports remains uncertain.
P03	Williams et al., 2019	Earlier reports and later Scaglione/Horvath cohorts when matching details were available	Williams overview patient; source family/pedigree label when reported	As reported in source overview; genotype group summarized in Supplementary Table 1	Age/sex not uniformly extractable from overlapping summaries	The Williams overview could not be matched patient by patient with all earlier or later reports because individual case details were limited.	Included among the 34 patients summarized by Williams et al.; possible overlap with other reports remains uncertain.
P04	Williams et al., 2019	Earlier reports and later Scaglione/Horvath cohorts when matching details were available	Williams overview patient; source family/pedigree label when reported	As reported in source overview; genotype group summarized in Supplementary Table 1	Age/sex not uniformly extractable from overlapping summaries	The Williams overview could not be matched patient by patient with all earlier or later reports because individual case details were limited.	Included among the 34 patients summarized by Williams et al.; possible overlap with other reports remains uncertain.
P05	Williams et al., 2019	Earlier reports and later Scaglione/Horvath cohorts when matching details were available	Williams overview patient; source family/pedigree label when reported	As reported in source overview; genotype group summarized in Supplementary Table 1	Age/sex not uniformly extractable from overlapping summaries	The Williams overview could not be matched patient by patient with all earlier or later reports because individual case details were limited.	Included among the 34 patients summarized by Williams et al.; possible overlap with other reports remains uncertain.
P06	Williams et al., 2019	Earlier reports and later Scaglione/Horvath cohorts when matching details were available	Williams overview patient; source family/pedigree label when reported	As reported in source overview; genotype group summarized in Supplementary Table 1	Age/sex not uniformly extractable from overlapping summaries	The Williams overview could not be matched patient by patient with all earlier or later reports because individual case details were limited.	Included among the 34 patients summarized by Williams et al.; possible overlap with other reports remains uncertain.
P07	Williams et al., 2019	Earlier reports and later Scaglione/Horvath cohorts when matching details were available	Williams overview patient; source family/pedigree label when reported	As reported in source overview; genotype group summarized in Supplementary Table 1	Age/sex not uniformly extractable from overlapping summaries	The Williams overview could not be matched patient by patient with all earlier or later reports because individual case details were limited.	Included among the 34 patients summarized by Williams et al.; possible overlap with other reports remains uncertain.
P08	Williams et al., 2019	Earlier reports and later	Williams overview	As reported in source	Age/sex not uniformly	The Williams overview could not be matched	Included among

Review ID	Primary publication	Overlapping/follow-up publications	Family/pedigree identifier	Genotype	Sex / age information	Information suggesting the same patient	Interpretation
		Scaglione/Horvath cohorts when matching details were available	patient; source family/pedigree label when reported	overview; genotype group summarized in Supplementary Table 1	extractable from overlapping summaries	patient by patient with all earlier or later reports because individual case details were limited.	the 34 patients summarized by Williams et al.; possible overlap with other reports remains uncertain.
P09	Williams et al., 2019	Earlier reports and later Scaglione/Horvath cohorts when matching details were available	Williams overview patient; source family/pedigree label when reported	As reported in source overview; genotype group summarized in Supplementary Table 1	Age/sex not uniformly extractable from overlapping summaries	The Williams overview could not be matched patient by patient with all earlier or later reports because individual case details were limited.	Included among the 34 patients summarized by Williams et al.; possible overlap with other reports remains uncertain.
P10	Williams et al., 2019	Earlier reports and later Scaglione/Horvath cohorts when matching details were available	Williams overview patient; source family/pedigree label when reported	As reported in source overview; genotype group summarized in Supplementary Table 1	Age/sex not uniformly extractable from overlapping summaries	The Williams overview could not be matched patient by patient with all earlier or later reports because individual case details were limited.	Included among the 34 patients summarized by Williams et al.; possible overlap with other reports remains uncertain.
P11	Williams et al., 2019	Earlier reports and later Scaglione/Horvath cohorts when matching details were available	Williams overview patient; source family/pedigree label when reported	As reported in source overview; genotype group summarized in Supplementary Table 1	Age/sex not uniformly extractable from overlapping summaries	The Williams overview could not be matched patient by patient with all earlier or later reports because individual case details were limited.	Included among the 34 patients summarized by Williams et al.; possible overlap with other reports remains uncertain.
P12	Williams et al., 2019	Earlier reports and later Scaglione/Horvath cohorts when matching details were available	Williams overview patient; source family/pedigree label when reported	As reported in source overview; genotype group summarized in Supplementary Table 1	Age/sex not uniformly extractable from overlapping summaries	The Williams overview could not be matched patient by patient with all earlier or later reports because individual case details were limited.	Included among the 34 patients summarized by Williams et al.; possible overlap with other reports remains uncertain.
P13	Williams et al., 2019	Earlier reports and later Scaglione/Horvath cohorts when matching details were available	Williams overview patient; source family/pedigree label when reported	As reported in source overview; genotype group summarized in Supplementary Table 1	Age/sex not uniformly extractable from overlapping summaries	The Williams overview could not be matched patient by patient with all earlier or later reports because individual case details were limited.	Included among the 34 patients summarized by Williams et al.; possible overlap with other reports remains uncertain.
P14	Williams et al., 2019	Earlier reports and later Scaglione/Horvath cohorts when matching details were available	Williams overview patient; source family/pedigree label when reported	As reported in source overview; genotype group summarized in Supplementary Table 1	Age/sex not uniformly extractable from overlapping summaries	The Williams overview could not be matched patient by patient with all earlier or later reports because individual case details were limited.	Included among the 34 patients summarized by Williams et al.; possible overlap with other reports remains uncertain.
P15	Williams et al., 2019	Earlier reports and later Scaglione/Horvath cohorts when matching details were available	Williams overview patient; source family/pedigree label when reported	As reported in source overview; genotype group summarized in Supplementary Table 1	Age/sex not uniformly extractable from overlapping summaries	The Williams overview could not be matched patient by patient with all earlier or later reports because individual case details were limited.	Included among the 34 patients summarized by Williams et al.; possible overlap with other reports remains uncertain.
P16	Williams et al., 2019	Earlier reports and later Scaglione/Horvath cohorts when matching details were available	Williams overview patient; source family/pedigree label when reported	As reported in source overview; genotype group summarized in Supplementary Table 1	Age/sex not uniformly extractable from overlapping summaries	The Williams overview could not be matched patient by patient with all earlier or later reports because individual case details were limited.	Included among the 34 patients summarized by Williams et al.; possible overlap with other reports

Review ID	Primary publication	Overlapping/follow-up publications	Family/pedigree identifier	Genotype	Sex / age information	Information suggesting the same patient	Interpretation
							remains uncertain.
P17	Williams et al., 2019	Earlier reports and later Scaglione/Horvath cohorts when matching details were available	Williams overview patient; source family/pedigree label when reported	As reported in source overview; genotype group summarized in Supplementary Table 1	Age/sex not uniformly extractable from overlapping summaries	The Williams overview could not be matched patient by patient with all earlier or later reports because individual case details were limited.	Included among the 34 patients summarized by Williams et al.; possible overlap with other reports remains uncertain.
P18	Williams et al., 2019	Earlier reports and later Scaglione/Horvath cohorts when matching details were available	Williams overview patient; source family/pedigree label when reported	As reported in source overview; genotype group summarized in Supplementary Table 1	Age/sex not uniformly extractable from overlapping summaries	The Williams overview could not be matched patient by patient with all earlier or later reports because individual case details were limited.	Included among the 34 patients summarized by Williams et al.; possible overlap with other reports remains uncertain.
P19	Williams et al., 2019	Earlier reports and later Scaglione/Horvath cohorts when matching details were available	Williams overview patient; source family/pedigree label when reported	As reported in source overview; genotype group summarized in Supplementary Table 1	Age/sex not uniformly extractable from overlapping summaries	The Williams overview could not be matched patient by patient with all earlier or later reports because individual case details were limited.	Included among the 34 patients summarized by Williams et al.; possible overlap with other reports remains uncertain.
P20	Williams et al., 2019	Earlier reports and later Scaglione/Horvath cohorts when matching details were available	Williams overview patient; source family/pedigree label when reported	As reported in source overview; genotype group summarized in Supplementary Table 1	Age/sex not uniformly extractable from overlapping summaries	The Williams overview could not be matched patient by patient with all earlier or later reports because individual case details were limited.	Included among the 34 patients summarized by Williams et al.; possible overlap with other reports remains uncertain.
P21	Williams et al., 2019	Earlier reports and later Scaglione/Horvath cohorts when matching details were available	Williams overview patient; source family/pedigree label when reported	As reported in source overview; genotype group summarized in Supplementary Table 1	Age/sex not uniformly extractable from overlapping summaries	The Williams overview could not be matched patient by patient with all earlier or later reports because individual case details were limited.	Included among the 34 patients summarized by Williams et al.; possible overlap with other reports remains uncertain.
P22	Williams et al., 2019	Earlier reports and later Scaglione/Horvath cohorts when matching details were available	Williams overview patient; source family/pedigree label when reported	As reported in source overview; genotype group summarized in Supplementary Table 1	Age/sex not uniformly extractable from overlapping summaries	The Williams overview could not be matched patient by patient with all earlier or later reports because individual case details were limited.	Included among the 34 patients summarized by Williams et al.; possible overlap with other reports remains uncertain.
P23	Williams et al., 2019	Earlier reports and later Scaglione/Horvath cohorts when matching details were available	Williams overview patient; source family/pedigree label when reported	As reported in source overview; genotype group summarized in Supplementary Table 1	Age/sex not uniformly extractable from overlapping summaries	The Williams overview could not be matched patient by patient with all earlier or later reports because individual case details were limited.	Included among the 34 patients summarized by Williams et al.; possible overlap with other reports remains uncertain.
P24	Williams et al., 2019	Earlier reports and later Scaglione/Horvath cohorts when matching details were available	Williams overview patient; source family/pedigree label when reported	As reported in source overview; genotype group summarized in Supplementary Table 1	Age/sex not uniformly extractable from overlapping summaries	The Williams overview could not be matched patient by patient with all earlier or later reports because individual case details were limited.	Included among the 34 patients summarized by Williams et al.; possible overlap with other reports remains uncertain.
P25	Williams et al., 2019	Earlier reports and later Scaglione/Horvath cohorts when matching details were available	Williams overview patient; source family/pedigree label when reported	As reported in source overview; genotype group summarized in Supplementary Table 1	Age/sex not uniformly extractable from overlapping summaries	The Williams overview could not be matched patient by patient with all earlier or later reports because individual case details were limited.	Included among the 34 patients summarized by Williams et al.;

Review ID	Primary publication	Overlapping/follow-up publications	Family/pedigree identifier	Genotype	Sex / age information	Information suggesting the same patient	Interpretation
							possible overlap with other reports remains uncertain.
P26	Williams et al., 2019	Earlier reports and later Scaglione/Horvath cohorts when matching details were available	Williams overview patient; source family/pedigree label when reported	As reported in source overview; genotype group summarized in Supplementary Table 1	Age/sex not uniformly extractable from overlapping summaries	The Williams overview could not be matched patient by patient with all earlier or later reports because individual case details were limited.	Included among the 34 patients summarized by Williams et al.; possible overlap with other reports remains uncertain.
P27	Williams et al., 2019	Earlier reports and later Scaglione/Horvath cohorts when matching details were available	Williams overview patient; source family/pedigree label when reported	As reported in source overview; genotype group summarized in Supplementary Table 1	Age/sex not uniformly extractable from overlapping summaries	The Williams overview could not be matched patient by patient with all earlier or later reports because individual case details were limited.	Included among the 34 patients summarized by Williams et al.; possible overlap with other reports remains uncertain.
P28	Williams et al., 2019	Earlier reports and later Scaglione/Horvath cohorts when matching details were available	Williams overview patient; source family/pedigree label when reported	As reported in source overview; genotype group summarized in Supplementary Table 1	Age/sex not uniformly extractable from overlapping summaries	The Williams overview could not be matched patient by patient with all earlier or later reports because individual case details were limited.	Included among the 34 patients summarized by Williams et al.; possible overlap with other reports remains uncertain.
P29	Williams et al., 2019	Earlier reports and later Scaglione/Horvath cohorts when matching details were available	Williams overview patient; source family/pedigree label when reported	As reported in source overview; genotype group summarized in Supplementary Table 1	Age/sex not uniformly extractable from overlapping summaries	The Williams overview could not be matched patient by patient with all earlier or later reports because individual case details were limited.	Included among the 34 patients summarized by Williams et al.; possible overlap with other reports remains uncertain.
P30	Williams et al., 2019	Earlier reports and later Scaglione/Horvath cohorts when matching details were available	Williams overview patient; source family/pedigree label when reported	As reported in source overview; genotype group summarized in Supplementary Table 1	Age/sex not uniformly extractable from overlapping summaries	The Williams overview could not be matched patient by patient with all earlier or later reports because individual case details were limited.	Included among the 34 patients summarized by Williams et al.; possible overlap with other reports remains uncertain.
P31	Williams et al., 2019	Earlier reports and later Scaglione/Horvath cohorts when matching details were available	Williams overview patient; source family/pedigree label when reported	As reported in source overview; genotype group summarized in Supplementary Table 1	Age/sex not uniformly extractable from overlapping summaries	The Williams overview could not be matched patient by patient with all earlier or later reports because individual case details were limited.	Included among the 34 patients summarized by Williams et al.; possible overlap with other reports remains uncertain.
P32	Williams et al., 2019	Earlier reports and later Scaglione/Horvath cohorts when matching details were available	Williams overview patient; source family/pedigree label when reported	As reported in source overview; genotype group summarized in Supplementary Table 1	Age/sex not uniformly extractable from overlapping summaries	The Williams overview could not be matched patient by patient with all earlier or later reports because individual case details were limited.	Included among the 34 patients summarized by Williams et al.; possible overlap with other reports remains uncertain.
P33	Williams et al., 2019	Earlier reports and later Scaglione/Horvath cohorts when matching details were available	Williams overview patient; source family/pedigree label when reported	As reported in source overview; genotype group summarized in Supplementary Table 1	Age/sex not uniformly extractable from overlapping summaries	The Williams overview could not be matched patient by patient with all earlier or later reports because individual case details were limited.	Included among the 34 patients summarized by Williams et al.; possible overlap with other reports remains uncertain.
P34	Williams et al., 2019	Earlier reports and later Scaglione/Horvath cohorts	Williams overview patient; source	As reported in source overview; genotype group	Age/sex not uniformly extractable from	The Williams overview could not be matched patient by patient with all earlier or later	Included among the 34 patients

Review ID	Primary publication	Overlapping/follow-up publications	Family/pedigree identifier	Genotype	Sex / age information	Information suggesting the same patient	Interpretation
		when matching details were available	family/pedigree label when reported	summarized in Supplementary Table 1	overlapping summaries	reports because individual case details were limited.	summarized by Williams et al.; possible overlap with other reports remains uncertain.
P35	Xue et al., 2021	None identified as a duplicate	Chinese prenatal/neonatal case	c.462-2A>G + c.574C>T	Prenatal diagnosis; neonatal biochemical confirmation; sex not reported in extracted abstract	Unique compound-heterozygous prenatal/neonatal report	Considered a separate patient; based on the available clinical and molecular details.
P36	Lee-Barber et al., 2019	Williams et al., 2019 where applicable	US child	c.512_514delCCT + c.931G>T	Child; age/sex as reported in source	Acetaminophen-associated presentation and genotype used for matching	Considered a separate patient; based on the available clinical and molecular details.
P37	Lafci et al., 2021	Endocrine-focused literature reviews	Turkish patient	c.715C>G homozygous	Sex/age as reported in source	Distinct endocrine/renal presentation and genotype	Considered a separate patient; based on the available clinical and molecular details.
P38	Takaleh et al., 2024	Endocrine literature context	Case 1	As reported in source	Age/sex as reported in source	Case-level endocrine report; family structure reviewed	Considered a separate patient unless the source indicated otherwise; based on the available clinical and molecular details.
P39	Takaleh et al., 2024	Endocrine literature context	Case 2	As reported in source	Age/sex as reported in source	Case-level endocrine report; family structure reviewed	Considered a separate patient unless the source indicated otherwise; based on the available clinical and molecular details.
P40	Yildiz et al., 2025	Earlier online/ePub record and endocrine reviews	Sibling 1	As reported in source	Age/sex as reported in source	Sibling structure, genotype and endocrine phenotype reviewed	Considered a separate patient; based on the available clinical and molecular details.
P41	Yildiz et al., 2025	Earlier online/ePub record and endocrine reviews	Sibling 2	As reported in source	Age/sex as reported in source	Sibling structure, genotype and endocrine phenotype reviewed	Considered a separate patient; based on the available clinical and molecular details.
P42	Fallata et al., 2023	Saudi founder-genotype literature	Saudi patient with HPS	c.793del homozygous	8-year-old girl	Hepatopulmonary syndrome, Saudi origin, founder genotype and transplant evaluation	Considered a separate patient; based on the available clinical and molecular details.
P43	Atasever et al., 2025	LTx literature	Pediatric LTx recipient	As reported in source	Pediatric patient; age/sex as reported in source	Unique LTx indication/outcome not duplicated by earlier transplant cases	Considered a separate patient; based on the available clinical

Review ID	Primary publication	Overlapping/follow-up publications	Family/pedigree identifier	Genotype	Sex / age information	Information suggesting the same patient	Interpretation
							and molecular details.
P44	Asiri et al., 2025	None identified	Saudi girl	c.871_873delGAG p.(Glu291del) homozygous; reported as VUS/novel	9-month-old girl	Exact variant and focal liver lesion distinguished from Saudi founder cases	Included as patient 52 because the clinical picture strongly supported TALDO-D; the variant remains a VUS. Excluding this case gives n = 51.
P45	Galindo Marin et al., 2026	Adult late-onset literature	Spanish late-onset case	As reported in source	Adult; age/sex as reported in source	First Spanish case, late-onset presentation and source statement reviewed	Considered a separate patient unless the source indicated otherwise; based on the available clinical and molecular details.
P46	Horvath et al., 2026	Possible earlier regional reports considered	Adult patient 1	c.574C>T homozygous	Diagnosed at 17 years; neonatal hepatomegaly/cytopenias reported	Declared as newly reported adult patient by source	Considered a separate patient; based on the available clinical and molecular details.
P47	Horvath et al., 2026	Possible earlier regional reports considered	Adult patient 2	c.131_133del + exon 3 deletion	Diagnosed at 26 years; neonatal hepatomegaly/cytopenias reported	Declared as newly reported adult patient by source	Considered a separate patient; based on the available clinical and molecular details.
P48	Horvath et al., 2026	Dirim et al. considered where relevant	Adult patient 3	c.345dupA homozygous	Diagnosed at 32 years; neonatal hepatomegaly/cytopenias reported	Declared as newly reported adult patient by source	Considered a separate patient; based on the available clinical and molecular details.
P49	Koprulu and Er, 2026	Endocrine literature context	Premature ovarian insufficiency case	As reported in source	Age/sex as reported in source	Unique endocrine-focused adult/late-onset report compared with other endocrine cases	Considered a separate patient unless the source indicated otherwise; based on the available clinical and molecular details.
P50	Gurbanova et al., 2026	Nephrology literature context	Sibling with kidney disease	As reported in source	Age/sex as reported in source	Kidney phenotype and sibling structure reviewed	Considered a separate patient unless the source indicated otherwise; based on the available clinical and molecular details.
P51	Sumer Cosar et al., 2026	None identified	Turkish sibling 1	Novel homozygous *TALDO1* variant; exact HGVS not available in public abstract	Index case with infancy presentation; sex not reported in extracted abstract	Hepatosplenomegaly, elevated transaminases and fibrotic liver disease	Considered a separate patient; based on the available clinical and molecular details.
P52	Sumer Cosar et al., 2026	None identified	Turkish sibling 2	Novel homozygous *TALDO1* variant; exact	11-year-old sibling; sex not reported in	Family-screened sibling with chronic fibrotic liver disease and focal liver lesion	Considered a separate patient;

Review ID	Primary publication	Overlapping/follow-up publications	Family/pedigree identifier	Genotype	Sex / age information	Information suggesting the same patient	Interpretation
				HGVS not available in public abstract	extracted abstract		based on the available clinical and molecular details.
