# Supplementary material for: Liver Disease in Transaldolase Deficiency: Molecular Basis, Genotype–Phenotype Relationships, and Transplantation Outcomes—A Systematic Review
Source: Int J Mol Sci. 2026 Sep 11;27(18):8100. doi: 10.3390/ijms27188100 (PMC13606807; doi:10.3390/ijms27188100)
Supplement: Supplementary file 1 [file ijms-27-08100-s001.zip › TALDO_IJMS_Supplementary_Table_S5_JBI_appraisal.pdf]

Supplementary Table S5. Study-specific JBI-based assessment of reporting completeness.

This is a structured reporting-completeness appraisal, not a conventional risk-of-bias rating. Y, yes; N, no; U, unclear/not reported; NA, not applicable. For non-interventional diagnostic case reports, Q5–Q7 are NA unless a treatment/procedure and its outcomes were evaluated. For case series, non-consecutive retrospective or literature-derived samples are rated U for consecutive/complete inclusion. The appraisal does not address publication bias, duplicate reporting, confounding, or validity of pooled documentation counts.

Ref.	Report	Design	Tool	Q1	Q2	Q3	Q4	Q5	Q6	Q7	Q8	Q9	Q10	Overall	Main reporting limitations
1	Verhoeven 2001	case report	case report	Y	Y	Y	Y	NA	NA	NA	Y	NA	NA	High reporting completeness	Adequate diagnostic case description; treatment outcomes or adverse events not applicable or not reported.
2	Williams 2019	overview/case series	case series	Y	Y	Y	U	U	Y	Y	U	Y	Y	Moderate reporting completeness	Literature/overview cohort with overlap and heterogeneous follow-up.
3	Scaglione 2026	case series	case series	Y	Y	Y	U	U	Y	Y	Y	Y	Y	High reporting completeness	Detailed long-term cohort; overlap with earlier reports and non-consecutive/complete inclusion remain uncertain.
4	Horvath 2026	case series	case series	Y	Y	Y	U	U	Y	Y	Y	Y	Y	High reporting completeness	Detailed adult natural history; small selected series and uncertain completeness.
7	Verhoeven 2005	case report	case report	Y	Y	Y	Y	NA	NA	NA	Y	NA	NA	High reporting completeness	Adequate diagnostic case description; treatment outcomes or adverse events not applicable or not reported.
8	Valayannopoulos 2006	case series	case series	Y	Y	Y	U	U	Y	Y	U	Y	Y	Moderate reporting completeness	Clinical series described; consecutive/complete inclusion and uniform longitudinal follow-up unclear.
9	Wamelink 2008	case report	case report	Y	Y	Y	Y	NA	NA	NA	Y	NA	NA	High reporting completeness	Adequate diagnostic case description; treatment outcomes or adverse events not applicable or not reported.
10	Tylki-Szymanska 2009	case series	case series	Y	Y	Y	U	U	Y	Y	U	Y	Y	Moderate reporting completeness	Clinical series described; consecutive/complete inclusion and uniform longitudinal follow-up unclear.
11	Balasubramaniam 2011	case report	case report	Y	Y	Y	Y	NA	NA	NA	Y	NA	NA	High reporting completeness	Adequate diagnostic case description; treatment outcomes or adverse events not applicable or not reported.
12	Loeffen 2012	case series	case series	Y	Y	Y	U	U	Y	Y	U	Y	Y	Moderate reporting completeness	Clinical series described; consecutive/complete inclusion and uniform longitudinal follow-up unclear.
13	Leduc 2014	case report	case report	Y	Y	Y	Y	Y	Y	U	Y	NA	NA	High reporting completeness	LTx and biochemical outcome described; adverse-event reporting limited.
14	Tylki-Szymanska 2014	case series	case series	Y	Y	Y	U	U	Y	Y	U	Y	Y	Moderate reporting completeness	Clinical series described; consecutive/complete inclusion and uniform longitudinal follow-up unclear.
15	Al-Shamsi 2015	case	case	Y	Y	Y	U	U	Y	Y	U	Y	Y	Moderate	Clinical series described;

Ref.	Report	Design	Tool	Q1	Q2	Q3	Q4	Q5	Q6	Q7	Q8	Q9	Q10	Overall	Main reporting limitations
		series	series											reporting completeness	consecutive/complete inclusion and uniform longitudinal follow-up unclear.
16	Banne 2016	case report	case report	Y	Y	Y	Y	NA	NA	NA	Y	NA	NA	High reporting completeness	Adequate diagnostic case description; treatment outcomes or adverse events not applicable or not reported.
17	Rodan 2017	case report	case report	Y	Y	Y	Y	Y	Y	Y	Y	NA	NA	High reporting completeness	NAC intervention and follow-up reported; single case limits causal inference.
18	Lipinski 2018	case series	case series	Y	Y	Y	U	U	Y	Y	U	Y	Y	Moderate reporting completeness	Clinical series described; consecutive/complete inclusion and uniform longitudinal follow-up unclear.
20	Lee-Barber 2019	case report	case report	Y	Y	Y	Y	NA	NA	NA	Y	NA	NA	High reporting completeness	Adequate diagnostic case description; treatment outcomes or adverse events not applicable or not reported.
21	Lafci 2021	case report	case report	Y	Y	Y	Y	NA	NA	NA	Y	NA	NA	High reporting completeness	Adequate diagnostic case description; treatment outcomes or adverse events not applicable or not reported.
22	Stefanowicz 2021	case series	case series	Y	Y	Y	U	U	Y	Y	Y	Y	Y	High reporting completeness	Longitudinal transplant outcomes reported; small selected series.
23	Grammatikopoulos 2022	case series	case series	Y	Y	Y	U	U	Y	Y	Y	Y	Y	High reporting completeness	Transplant/HCC outcomes detailed; consecutive and complete inclusion unclear.
24	Fallata 2023	case report	case report	Y	Y	Y	Y	NA	NA	NA	Y	NA	NA	High reporting completeness	Transplant evaluation discussed; no completed intervention or post-LTx outcome.
25	Takaleh 2024	case series	case series	Y	Y	Y	U	U	Y	Y	U	Y	Y	Moderate reporting completeness	Clinical series described; consecutive/complete inclusion and uniform longitudinal follow-up unclear.
26	Yildiz 2025	case series	case series	Y	Y	Y	U	U	Y	Y	U	Y	Y	Moderate reporting completeness	Clinical series described; consecutive/complete inclusion and uniform longitudinal follow-up unclear.
27	Atasever 2025	case report	case report	Y	Y	Y	Y	Y	Y	Y	Y	NA	NA	High reporting completeness	LTx procedure, biliary complication, and one-year outcome reported.
28	Asiri 2025	case report	case report	Y	Y	Y	Y	NA	NA	NA	Y	NA	NA	High reporting completeness	Clinical and molecular description provided; variant remained VUS and biochemical confirmation was not reported.
29	Sumer Cosar 2026	case series	case series	Y	Y	Y	U	U	Y	Y	U	Y	Y	Moderate reporting completeness	Clinical series described; consecutive/complete inclusion and uniform longitudinal follow-up unclear.
30	Koprulu 2026	case report	case report	Y	Y	Y	Y	NA	NA	NA	Y	NA	NA	High reporting completeness	Adequate diagnostic case description; treatment outcomes or adverse events not applicable or not reported.
31	Gurbanova 2026	case	case	Y	Y	Y	U	U	Y	Y	U	Y	Y	Moderate	Clinical series described;

Ref.	Report	Design	Tool	Q1	Q2	Q3	Q4	Q5	Q6	Q7	Q8	Q9	Q10	Overall	Main reporting limitations
		series	series											reporting completeness	consecutive/complete inclusion and uniform longitudinal follow-up unclear.
32	Xue 2021	case report	case report	Y	Y	Y	Y	NA	NA	NA	Y	NA	NA	High reporting completeness	Adequate diagnostic case description; treatment outcomes or adverse events not applicable or not reported.
